# Tumor Necrosis Factor Receptor SF10A (TNFRSF10A) SNPs Correlate With Corticosteroid Response in Duchenne Muscular Dystrophy

**DOI:** 10.3389/fgene.2020.00605

**Published:** 2020-07-03

**Authors:** Chiara Passarelli, Rita Selvatici, Alberto Carrieri, Francesca Romana Di Raimo, Maria Sofia Falzarano, Fernanda Fortunato, Rachele Rossi, Volker Straub, Katie Bushby, Mojgan Reza, Irina Zharaieva, Adele D’Amico, Enrico Bertini, Luciano Merlini, Patrizia Sabatelli, Paola Borgiani, Giuseppe Novelli, Sonia Messina, Marika Pane, Eugenio Mercuri, Mireille Claustres, Sylvie Tuffery-Giraud, Annemieke Aartsma-Rus, Pietro Spitali, Peter A. C. T’Hoen, Hanns Lochmüller, Kristin Strandberg, Cristina Al-Khalili, Ekaterina Kotelnikova, Michael Lebowitz, Elena Schwartz, Francesco Muntoni, Chiara Scapoli, Alessandra Ferlini

**Affiliations:** ^1^Unit of Medical Genetics, Department of Medical Sciences, University of Ferrara, Ferrara, Italy; ^2^U.O.C. Laboratory of Medical Genetics, Paediatric Hospital Bambino Gesù, IRCCS, Rome, Italy; ^3^Department of Life Sciences and Biotechnology, University of Ferrara, Ferrara, Italy; ^4^John Walton Muscular Dystrophy Research Centre, Institute of Genetic Medicine, Newcastle University, Newcastle upon Tyne, United Kingdom; ^5^Dubowitz Neuromuscular Center, University College London Institute of Child Health & Great Ormond Street Hospital, London, United Kingdom; ^6^Molecular Medicine and Unit of Neuromuscular and Neurodegenerative Diseases, Paediatric Hospital Bambino Gesù, IRCCS, Rome, Italy; ^7^Department of Biomedical and Neuromotor Sciences, University of Bologna, Bologna, Italy; ^8^IRCCS Rizzoli & Institute of Molecular Genetics, National Research Council of Italy, Bologna, Italy; ^9^Genetics Unit, Department of Biomedicine and Prevention, University of Rome Tor Vergata, Rome, Italy; ^10^Istituto Neuromed, IRCCS, Pozzilli, Italy; ^11^Department of Clinical and Experimental Medicine, Nemo Sud Clinical Center, University of Messina, Messina, Italy; ^12^Paediatric Neurology Unit, Centro Clinico Nemo, IRCCS Fondazione Policlinico A. Gemelli, Universita’ Cattolica del Sacro Cuore, Rome, Italy; ^13^Laboratory of Genetics of Rare Diseases, University of Montpellier, Montpellier, France; ^14^Department of Human Genetics, Leiden University Medical Center, Leiden, Netherlands; ^15^Center for Molecular and Biomolecular Informatics, Radboud Institute for Molecular Life Sciences, Radboud University Medical Center, Nijmegen, Netherlands; ^16^Department of Neuropediatrics and Muscle Disorders, Faculty of Medicine, Medical Center – University of Freiburg, Freiburg, Germany; ^17^Centro Nacional de Análisis Genómico (CNAG-CRG), Center for Genomic Regulation, Barcelona Institute of Science and Technology (BIST), Barcelona, Spain; ^18^Children’s Hospital of Eastern Ontario Research Institute, Ottawa, ON, Canada; ^19^Division of Neurology, Department of Medicine, The Ottawa Hospital, Ottawa, ON, Canada; ^20^Brain and Mind Research Institute, University of Ottawa, Ottawa, ON, Canada; ^21^Department of Systems Biology, School of Chemistry, Biotechnology and Health, KTH – Royal Institute of Technology, Stockholm, Sweden; ^22^Panacea Pharmaceuticals, Gaithersburg, MD, United States; ^23^National Cancer Institute, Bethesda, MD, United States; ^24^NIH Great Ormond Street Hospital Biomedical Research Centre, Great Ormond Street Institute of Child Health, University College London, London, United Kingdom; ^25^Great Ormond Street Hospital Trust, London, United Kingdom

**Keywords:** biomarker, corticosteroid (betamethasone), receptor, TNFR, Duchenne

## Abstract

**Background:**

Duchenne muscular dystrophy (DMD) is a rare and severe X-linked muscular dystrophy in which the standard of care with variable outcome, also due to different drug response, is chronic off-label treatment with corticosteroids (CS). In order to search for SNP biomarkers for corticosteroid responsiveness, we genotyped variants across 205 DMD-related genes in patients with differential response to steroid treatment.

**Methods and Findings:**

We enrolled a total of 228 DMD patients with identified dystrophin mutations, 78 of these patients have been under corticosteroid treatment for at least 5 years. DMD patients were defined as high responders (HR) if they had maintained the ability to walk after 15 years of age and low responders (LR) for those who had lost ambulation before the age of 10 despite corticosteroid therapy. Based on interactome mapping, we prioritized 205 genes and sequenced them in 21 DMD patients (discovery cohort or DiC = 21). We identified 43 SNPs that discriminate between HR and LR. Discriminant Analysis of Principal Components (DAPC) prioritized 2 response-associated SNPs in the *TNFRSF10A* gene. Validation of this genotype was done in two additional larger cohorts composed of 46 DMD patients on corticosteroid therapy (validation cohorts or VaC1), and 150 non ambulant DMD patients and never treated with corticosteroids (VaC2). SNP analysis in all validation cohorts (*N* = 207) showed that the CT haplotype is significantly associated with HR DMDs confirming the discovery results.

**Conclusion:**

We have shown that TNFRSF10A CT haplotype correlates with corticosteroid response in DMD patients and propose it as an exploratory CS response biomarker.

## Introduction

Duchenne muscular dystrophy (DMD, OMIM ^∗^310200) is a rare hereditary disease due to mutations in the dystrophin (*DMD)* gene, which maps to the X-chromosome (Xp21.1), and affects 1 in 5,000 newborn males. It is characterized by the almost complete absence of the dystrophin protein (DYS) in muscle fibers, which causes progressive muscle damage leading to death in the first 3 decades of life (Goemans and Buyse, 2014). Glucocorticosteroids (CS) have been demonstrated to be effective in delaying the progression of this illness. Two decades of randomized clinical trials on large DMD cohorts using various treatment regimens have shown that CS use increases muscle strength and delays loss of ambulation (LoA), progression of respiratory dysfunction, dilated cardiomyopathy and onset of scoliosis (Bushby et al., 2010; Griggs et al., 2016). CS use is part of the DMD standards of care (Bushby et al., 2010), but were used off-label. Recently, the Food and Drug Administration (FDA, United States) approved the CS Emiflaza (deflazacort) for the indication of DMD^1^. Since this approval, CSs are now used as an approved orphan drug for DMD patients in the United States.

Although CS have been shown to be beneficial for many multisystemic complications of DMD, they cannot recover prior lost function, therefore some authors suggest that treatment with CS should begin early in the course of the disease (Merlini et al., 2003). The two common regimens are daily and intermittent (10 days on, 10 days off) CS administration (Bushby et al., 2010; Griggs et al., 2016). The anti-inflammatory properties of CS, mediated predominantly through monomer CS or glucocorticoid receptor (GR) inhibition of transcription factors such as NF-kB (transrepression) are considered important in DMD therapy. To exert their effects, CS bind the GR, which is a ligand-induced transcription factor belonging to the nuclear hormone family. When not bound to hormones, GR resides in the cytoplasm, sequestered by heat shock proteins. GR mediates a number of other effects using many tethered interactions both at the DNA level, binding CS response elements (including one recently identified within the *DMD* gene) (Wein et al., 2014) and by recruiting other transcription factors and proteins. All these actions point toward a transcriptional process that is highly dynamic, including chromatin remodeling, and depend on cell and tissue types. Nevertheless, the pharmacodynamics regulation of CS is not completely deciphered (Miranda et al., 2013; Whirledge and DeFranco, 2018).

Not all DMD patients tolerate chronic use of CS and treatment often has to be stopped or dosage substantially reduced to mitigate adverse effects in a subset of patients; in addition, not all DMD individuals have the same beneficial response to CS therapy (McDonald et al., 2018). Therefore, in view of chronic treatment-related severe side effects, personalized treatment plans would be preferred. Several studies have focused on identifying genetic variants that impact the efficacy of CS treatment in various pathologies, and two SNPs in the corticotrophin-releasing hormone receptor 1 (*CRHR1*) and in the glucocorticoid-induced transcript 1 (*GLCCI1*) genes, have been identified. These SNPs have already been explored to validate pharmacogenetic biomarkers to CS response in asthma (Tantisira et al., 2004, 2011; Levin et al., 2018) and were suggested as exploratory in DMD (Bonifati et al., 2006), but not further confirmed. The advent of next generation sequencing (NGS) strategies, and the resulting data deciphered and interpreted using novel bioinformatics tools, has allowed researchers to carry out massive sequence analysis on several genes in order to identify candidate SNPs, which may play a role in determining disease aetiology, status, progression risk, disease modifiers, and response to drugs (Jombart and Ahmed, 2011; Kotelnikova et al., 2012).

SNPs associated with DMD muscle performance, especially ambulation loss, have already been described in a few papers (Flanigan et al., 2013; Bello et al., 2015; Ferlini et al., 2015; van den Bergen et al., 2015; Vo and McNally, 2015; Szigyarto and Spitali, 2018). Nevertheless, robust data on SNP biomarkers specifically linked to corticosteroid response in DMD are lacking. In order to identify SNP possibly linked to CS response in DMD boys, we studied a total of 217 DMD patients and defined a high responder (HR) subgroup in patients who had maintained the ability to walk after 15 years (from 16 and on) and low responder (LR) subgroup for the DMD boys who had lost ambulation before the age of 10 despite of CS therapy. These DMDs were divided in three cohorts. The first cohort (Discovery cohorts – abbreviated as DiC) was composed of 21 DMDs all on CS therapy and it was used for NGS-based biomarker discovery. The other 2 cohorts were used as validation cohorts (VaC1, VaC2). We were able to prioritize two SNPs (causing the missense variations p.His141Arg and p.Arg209Thr) in the *TNFRSF10A* gene coding sequence. Validation of these SNPs was carried out in the validation cohort VaC1, composed of 46 patients under CS treatment and in validation cohort VaC2 composed of 150 patients never treated with CS.

Two-dimension statistical analysis suggested that *TNFRSF10A* C/T haplotype is associated with HR patients. We suggest that TNFRSF10A is a good candidate pharmacogenetics biomarker for CS response in DMD.

## Materials and Methods

### Patient Enrollment

We enrolled 217 DMD patients in total. The study was conducted within the BIO-NMD project ethical approval at the Ferrara University Ethical Committee (N. 11/2010).

The diagnosis of DMD was made based on established standard clinical outcome measures and scales and *DMD* mutation (Bushby et al., 2010). The clinical assessment included age of onset and disease progression. Patients were defined as belonging to one of two diagnostic classes by age of LoA: the DMD boys were defined as low responders (LoA before age 10) and high responders (LoA after age 15), both on corticosteroid treatment for at least 24 months (Ricotti et al., 2016; McDonald et al., 2017). In VaC2 (150 patients), DMDs were non-ambulant and never treated with CS treatment.

We selected patients under CS from at least 24 months, based on the available clinical information. It is possible that also the duration of CS treatment may play a role in giving different clinical outcomes in terms of expected age of LoA. Introducing an additional parameter (duration in years) would have further reduced the number of patients to be enrolled in this study, possibly further reducing the statistical power of our study.

The enrolled patients were divided into three cohorts: the targeted sequencing discovery cohort (DiC) composed of 21 DMDs all CS treated (13 low responders and 8 high responders). In all 21 patients, targeted sequencing was performed by Solid platform. The Validation Cohorts are composed of two DMD populations: the BIO-NMD cohorts (VaC1 *N* = 46) including 46 DMDs, all CS treated, of which 26 are low responders and 20 high responders, and selected by the identical criteria adopted for the DiC; the DMD cohort (VaC2 *N* = 150) never treated with corticosteroids (Bushby et al., 2010). These 150 non-ambulant DMD patients were considered as validation cohort since their ambulation status was certainly unrelated to the CS therapy.

Table 1 describes the summary of DMD cohorts enrolled for the study (A) and the clinical and genetic features of DMD patients in the DiC (B). Patients in the DiC were enrolled in NewCastle Center (via the EuroBiobank, Newcastle & North Tyneside 1 Research Ethics Committee number: 19/NE/0028 and Newcastle upon Tyne Hospitals R&D Number: 9182), the UCL Center (Biobank Research Ethics Committee number REC Reference: 06/Q0406/33)^2^, and the UNIFE Center (Area Vasta Centrale Bologna Ethical Committee approval N. 11/2010).

**TABLE 1:** Patients’ cohorts description.

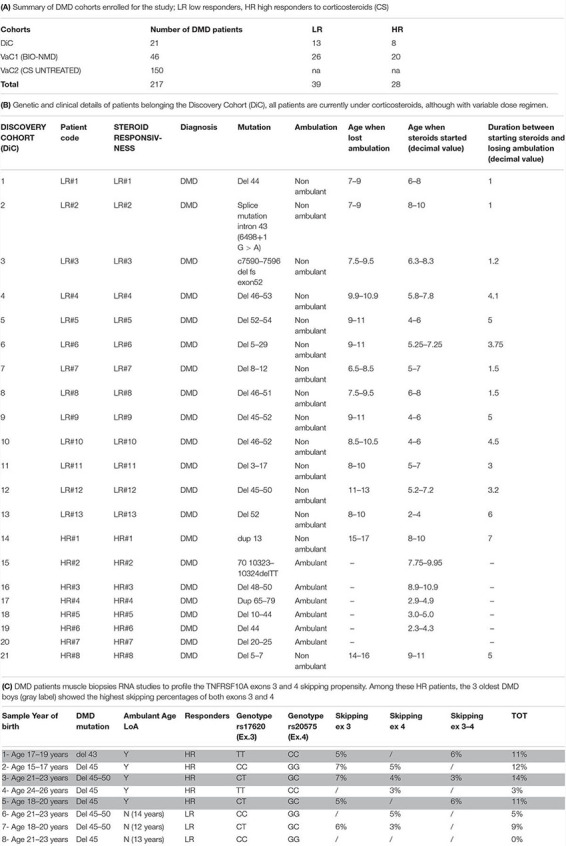

The informed consents obtained were collected according to the local ethical rules of the centers, above reported.

We also analyzed muscle biopsies from additional 8 patients with different CS response to evaluate the TNFRSF10A exons 3 and 4 skipping propensity (Table 1C) via UNIFE under the ethical rules above cited for the other UNIFE patients.

Patients age when lost ambulation and age when steroids started (decimal value) (Table 1B) and ages (Table 1C) have been reported as an average accordingly to the ethical rules not allowing indirect patients data identification. True values can be obtained by simply calculating the mean values.

### Selection Criteria to Prioritize the NMD Database Genes

The final priority listing of DMD-associated 884 genes proposed in the BIO-NMD project^3^ and listed in Kotelnikova et al. (2012), was used to identify overall DMD-associated genes/proteins. The following factors were considered in the biomarker prioritization scheme: (1) Experimental Evidence: if evidence supporting the association of a gene with DMD was obtained in an independent experiment (immunoassay, 2D-DIGE, expression studies) within the BIO-NMD project, the gene was scored as a “1”; thus this parameter simply counted the number of independent experiments that pointed to the association of a particular gene with DMD; (2) Pathway Information: the presence of a gene/protein existing in manually curated pathways, cellular processes and/or Gene Ontology terms related to muscular dystrophy was taken into account and afforded weight in analyzing the importance of the gene’s association with DMD; (3) Literature References: the number of citations in the existing literature (updated at September 2016) linking this gene/protein to DMD was also considered and the total number of citations in the existing literature (at September 2016) linking this gene/protein to muscular disease in general was also considered; (4) Protein Characteristics/Ease of Analysis: the information concerning the protein role (structural, regulatory, biochemical) was considered in the prioritization scheme. Also, because some of the identified genes that were identified in animal model experiments or in the pathway analysis do not have the human counterparts, whether or not the gene or protein had been previously reported in humans was also considered. Values for each parameter were normalized on a scale of 0–1, where 1 is assigned to the maximum value for that parameter. Each parameter was then multiplied by the given weight and the weighted scores were summed.

The top 205 DMD-associated genes out of a total of 884 genes were thus identified by at least one experiment performed within the BIO-NMD consortium (Ferlini et al., 2015). The 205 prioritized genes used to design the sequencing array are listed in Supplementary Table S1. Notably, neither CRHR1 and GLCC11 were selected by prioritization.

### SOLiD Targeted Resequencing and SNPs Validation

We developed a workflow based on NGS using the 5500 SOLiD Sequencer (Life Technologies) for the identification of SNPs in the selected patient samples. For the specific interrogation of regions of interest (ROI), we conducted targeted sequencing of the exons of genes, which are connected to the DMD pathway and preceded by a targeted specific enrichment step. For target enrichment, Agilent’s SureSelect in solution approach was selected. Based on the target gene lists, the panel of capture probes was designed using the Agilent eArray tool (see the 205 enriched gene list in Supplementary Table S1). Libraries were sequenced with 75 bp single end reads. The DiC patients were run on the 5500xl SOLiD^TM^ Sequencer. Mapping, as well as SNP and indel calling, were carried out using the LifeScope 2.5^4^.

Quality filtering was carried out on the SNP calls returned by LifeScope using quality thresholds for base quality values and coverage, as well as rule-based filtering for exclusion of PCR artifacts, strand bias and quality imbalances between called alleles. We have considered SNPs located in the coding regions only since the functional meaning of non-coding region SNPs is very difficult to predict since not present in ExAC database^5^.

The 21 DMD patients in the DiCs and the 46 patients in the VaC1 were further genotyped by Sanger for the *TNFRSF10A* SNPs (to validate the SOLiD data) and for VCAN, LTBP4, SPP1, GLCCI2, and CRHR1 SNPs (the oligonucleotides used are in Supplementary Table S2A). Polymerase chain reaction (30 cycles) was performed on genomic DNA using specific pairs of primers and all PCRs were run in a final volume of 25 μl, containing 100 ng genomic DNA, 20 mM Tris–HCl (pH 8.4), 50 mM KCl, 1.5 mmol/l MgCl_2_, 0.2 mmol/l dNTPs, 5U Taq DNA Polymerase (Invitrogen) and 0.4 μl of each primer.

The VaC2 DMD patients (*N* = 150) were *TNFRSF10A* genotyped using the Sequenom MassARRAY^®^ platform (Inc., San Diego, CA, United States) according to manufacturer’s protocols except for the PCR protocol, which was a step-down protocol. Genotyping assays were designed using Sequenom MassARRAY Assay^®^ Design Suite, version 1.0 (Sequenom Inc.). After the primer extension reaction, the products were spotted onto a target chip with 384 patches containing matrix. Mass differences were detected using the MassARRAY^®^ Compact System (Bruker, Wormer, Netherlands) by MALDI-TOF. Data were acquired with the MassARRAY^®^ RT software (Sequenom Inc.) and genotypes were assigned using MassARRAY Typer 4.0.22 software (Sequenom Inc.).

### SNPs Prioritization and Association Statistical Analysis

In order to prioritize the SNPs identified by target gene resequencing in the 205 genes, the exploratory Discriminant Analysis of Principal Components (DAPC) was applied using the Adegenet package (Jombart and Ahmed, 2011) (function dapc) for software R (R Development Core Team, 2011).

DAPC is based on data transformation, which ensures that variables submitted to DA are perfectly uncorrelated and that their number is less than that of the analyzed individuals. DAPC first performs a PCA, identifying the directions of maximal variance, then the most informative directions (PC’s) are picked and a K-means clustering is performed on the data in order to maximize the variation between K groups by incrementally increasing K. In this study, DAPC defines a model in which genetic variation is partitioned into a “between-” and a “within-” group component, and yields synthetic variables (i.e., SNPs), which maximize the first while minimizing the second.

The analysis was performed with and without prior information on individual populations. In the second analysis, the number of clusters was assessed using the *find clusters* function, which runs successive K-means clustering with increasing number of clusters (k). For selecting the optimal number of clusters, we applied the Bayesian Information Criterion (BIC) for assessing the best supported model, and therefore the number and nature of clusters, as recommended by Chadeau-Hyam et al. (2013).

The associations of validated gene polymorphisms in *TNFRSF10A* and different responses to therapy (HR vs. LR patients in both the DiC and VaCs) were performed by comparing genotypic/allelic distributions in HR/LR subjects, through the maximum likelihood chi square based on the additive model (ML χ^2^) estimated by log-linear analysis as implemented in Statistica Package (STATISTICA 7.1, StatSoft, Inc., Tulsa, OK, United States). For all data analysis, significance level was set at 5%. The *p*-values in Table 2 remain significant even after the Bonferroni Correction.

**TABLE 2:** SNPs analysis results in all patients cohorts. **(A-1)**

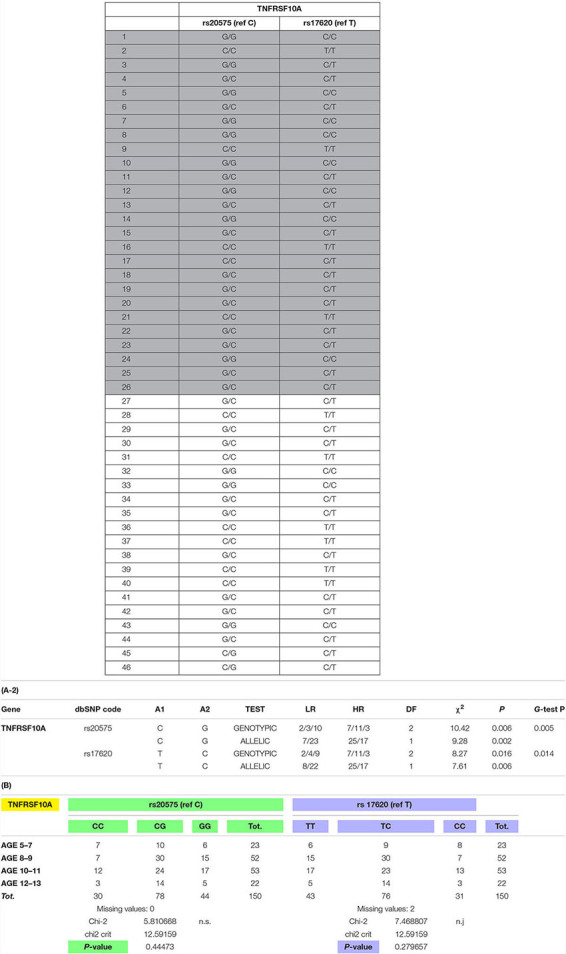

***(A)** Validation of the TNFRSF10A SNPs in the VaC1. (1) VaC1 (46 DMD patients): Distribution of TNFRS10A genotypes in LR and HR in 46 VaC DMD patients (LOW (1–26) AND HIGH (27–46) RESPONDERS). (2) Comparison between allelic and genotypic frequencies of TNFRSF10A variants in Low Responders (LR) and High Responders (HR); RefSNP Alleles (A1, A2 where A1 is the ancestral allele); contingency χ^2^ (Pearson Uncorrected) and G test probability value (P). **(B)** Analysis of prioritized TNFRSF10A SNPs in the VaC2/French patients steroids naïve (never treated by corticosteroids) for whom age at loss of ambulation cannot be related to the corticosteroid effect. Genotype and haplotype are showed, refseq of the SNPs are reported. Alleles are stratified by age, and p value was calculated by comparing allelic distributions in different age categories, through the maximum likelihood chi square based on the additive model. The Sequenom MassARRAY analysis revealed no association between SNPs with the age of loss of ambulation.*

### TNFRSF10A SNPs Effect on Transcript Splicing

RNA from the 8 DMD muscle biopsies (Table 1C) were extracted and reverse-transcribed as described in Bovolenta et al. (2012).

Exon skipping was quantified by an Agilent 2100 bioanalyzer (Agilent Technologies, Santa Clara, CA, United States). The region spanning exons 2–7 of TNFRSF10A was amplified, and PCRs were performed via 35 cycles at 94°C (30 s), 60°C (45 s), and 72°C (80 s), with Invitrogen Platinum Taq DNA polymerase (Thermo Fisher Scientific). PCR products were analyzed with the Agilent high-sensitivity DNA chip in order to measure both DNA concentration and size. The skipping percentages were calculated as the ratio between skipped transcript and total transcript. Exon skipping % = (molarity skipped transcripts)/(molarity skipped transcripts + molarity non-skipped transcripts) × 100%.

### TNFRSF10A Immunoassay

TNFRSF10A immunoassay was performed on 12 HR and 11 LR plasma and serum samples.

Two validated anti-TNFRSF10A antibodies (western blot, immunohistochemical staining, and protein arrays) recognizing aa 32–66 and 105–139, respectively, were selected for proteomics (Uhlén et al., 2015). Briefly 1.75 μg of each antibody were coupled to color-coded, carboxylated magnetic beads (MagPlex, Luminex Corporation) according to previously established protocol (Ayoglu et al., 2014). The coupling efficiency was determined by incubation with R-phycoerythrin (R-PE) conjugated anti-rabbit antibodies. Genetically confirmed and diagnosed DMD patient plasma samples collected within the BIO-NMD consortium from University College of London (UCL), London, United Kingdom were analyzed. Three microliter of sample were biotinylated and used for the analysis of TNFRSF10A (Ayoglu et al., 2014).

### Bioinformatics Tools

We used the Pathway Studio 9.0 from Elsevier for SNEA analysis as described before (Kotelnikova et al., 2012). This method identifies subnetworks containing a central regulator (including but not limited to transcription factors) and downstream target genes, which have significantly co-operatively changed their expression. The algorithm starts with selecting the central “seed” from one of the relevant entities (protein, complex, or set of proteins, “functional class”) in the database. The database (called Resnet) stores literature-extracted biomedical entities and their relations. SNEA creates a subnetwork by retrieving all entities interacting with the selected seed. We used two types of interactions – Expression (300465 relations in Resnet) and PromoterBinding (18153 relations in Resnet). The next algorithm uses the Mann-Whitney *U*-test to calculate the *p*-value for differences between distribution of expression values of the regulator’s downstream genes and background distribution of all expression values for the selected sample in the experiment. In order to correct for biases introduced by hubs, the expression value for each entity connected to a seed is accounted for as many times as the connectivity of that entity in ResNet during distribution calculation. Finally, subnetworks are ranked according to *P*-values and the top 100 subnetworks with a p-value smaller than 0.05 are returned by default.

For the pie chart analysis we used the *Functional Enrichment analysis tool*^6^ and the UniProt database.

## Results

### Targeted Genes Resequencing

We sequenced the 205 prioritized genes by SOLiD sequencing in 21 DMD patients belonging to the DiC and SNP calling and retrieved a total of 1714 SNPs exonic variants in all the lanes. We selected only SNPs already present in dbSNP because we were looking for discriminant variants (medium/high allele frequency in the database) and not for rare variations/disease causing mutations. Overall, 595 SNPs (34.7% of the total) were present in the dbSNP. We then excluded variants with MAF < 0.05 and filtered both SNPs and samples for a CallRate > 90%. Thus, we called 354 SNPs, 220 synonymous and 134 non-synonymous, in the 21 analyzed DMD patients. All the 354 SNPs passed the quality control filters. Supplementary Table S3 lists the 354 SNPs called.

### Statistical Analysis, SNPs Prioritization, and Validation

**FIGURE 1:**
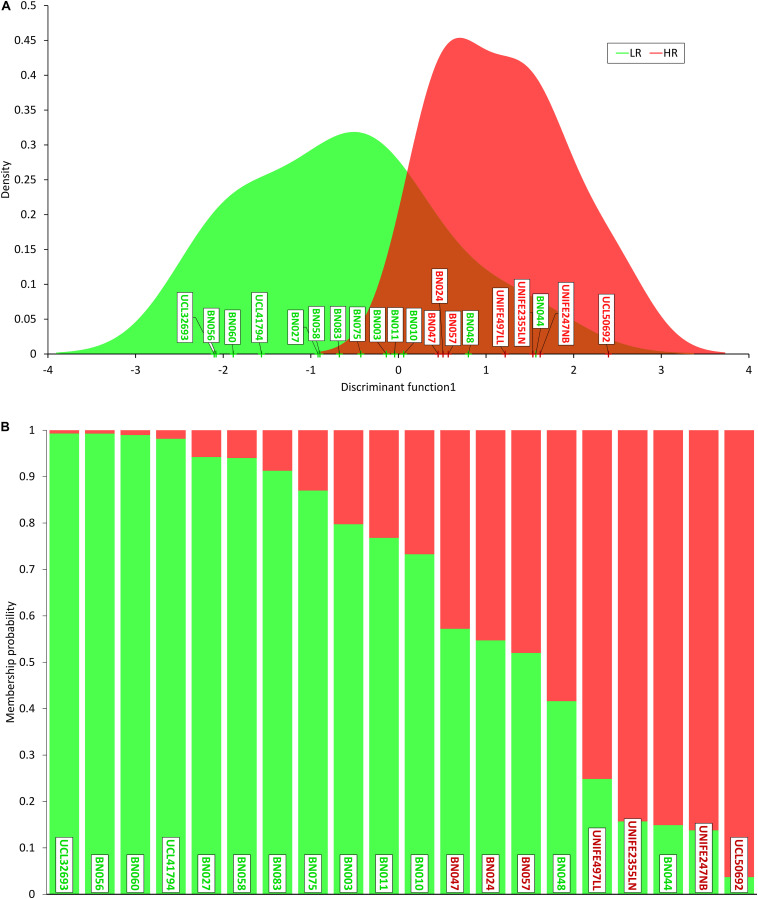
Discriminant analysis of principal components (DAPC). **(A)** Density of individual scores on the first discriminant function, low responders (LR) in green and high responders (HR) in red; **(B)** membership probability (assignment) of individuals to the two groups based on the retained discriminant functions. Each individual is represented as a vertical bar, where colors corresponding to probabilities of membership to LR (green) and HR (red). Note that three HR patients show a higher “genetic proximity” to LR cluster and two LR subjects are assigned to HR cluster.

In order to prioritize the 354 SNPs identified by sequencing, we performed DAPC to identify and describe clusters of genetically related individuals (Jombart et al., 2010; Jombart and Ahmed, 2011). DAPC was performed on both the whole set of 354 SNPs identified in the 205 genes sequenced and separately on 220 synonymous and 134 non-synonymous variants. The best discrimination among patients was obtained in the latter group (the non-synonymous being more likely effective) with a proportion of variance (explained by PC1) of 51.3% (Supplementary Table S1 and Figure 1).

**FIGURE 2:**
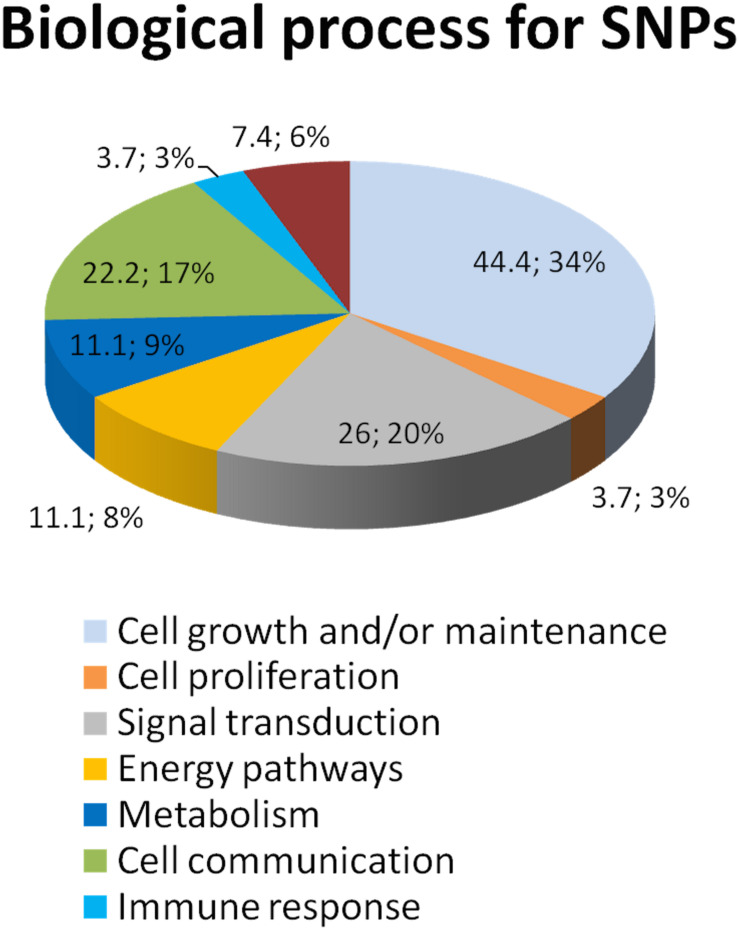
Ontology group analysis revealed that SNPs resulting from DAPC analysis belong to several biological processes. We utilized the Sub-Network Enrichment Analysis (SNEA) to determine the possible pathways that are responsible for the CS response. SNEA is based on the Gene Set Enrichment Analysis algorithm. Sub-Networks of the potential pathways that regulate CS response are calculated *de novo* from the information in the data-sets and consist of a seed/regulator and their neighbors (targets) in the database. The seeds of the sub-network whose targets are statistically enriched are implicated as important regulators (cell processes) by the experimental data. The data shows that these 43 prioritized SNPs are involved in the pathways regulate cytokines, GR signaling, the TNF-induced cytotoxicity, and many others.

DAPC analysis sorted out 21 non-synonymous and 22 synonymous SNPs (Supplementary Table S4). Ontology group analysis on these 43 prioritized SNPs revealed that they belong to many circuits, of which major representation is visible on cell growth maintenance and signaling (Figure 2).

On the basis of highest contributions of original variables (alleles) to the principal components of DAPC, we prioritized 4 SNPs (two synonymous and two missense) in 2 genes: *VCAN* (rs4470745 and rs12332199) for synonymous and *TNFRSF10A* (rs20575 and rs17620) for missense. We technically validated these 4 SNPs by resequencing these 4 variants with an independent technology in the DiC cohort and further analyzed these 4 SNPs in the validation cohorts (VaC1 by Sanger and VaC2 by Sequenom). We obtained informative significant results both for allelic and genotypic distributions for *TNFRSF10A* only, and the C and T alleles (C/T haplotype) seem to confer a better response to CS (Table 2). *VCAN* gene SNPs were not significant in all the VaCs (not shown).

In the VaC2 cohort, we also tested the relationship between *TNFRSF10A* SNPs and the age of LoA, however, no association between these variables was found. Since the LoA in these 150 patients was not influenced by CS therapy, *TNFRSF10A* therefore seems associated with the maintenance of ambulation only in patients under CS therapy and therefore mainly related to CS response. The results of TNFRSF10A SNP analysis are shown in Table 2.

Finally in order to evaluate previously reported results, we genotyped *LTBP4*, *SPP1*, *CRHR1*, and *GLCCI1* in DiC and VaC1. The association tests (Uncorrected Pearson X ^2^ and *G*-test) according to HR and LR phenotypes showed an association with CS response only for SNPs in the *LTBP4* gene for which we obtained nominal significant levels of association for genotypes but not for allelic distributions. All other tested SNPs (*SPP1*, *CRHR1*, and *GLCCI1)* showed negative results (Supplementary Table S2B).

Supplementary Figure S1 shows the *TNFRSF10A* gene architecture and the mapping of the two SNPs rs17620 and rs20575. These SNPs cause the missense variations H141R and R209T, predicted as benign and damaging, respectively, by Polyphen. Interestingly, both missense variations are located in the Cystein-rich TNFR extracellular domains 1 and 2. The receptor’s extracellular cysteine-rich domain (1–3) contains the ligand area deputed to bind Trail-1 (Guicciardi and Gores, 2009). Therefore, missense variations in this region may influence ligand-receptor interactions and subsequent apoptosis-related functions and may affect the regeneration capacity of the cell.

### TNFRSF10A SNPs Effect on Splicing

**FIGURE 3:**
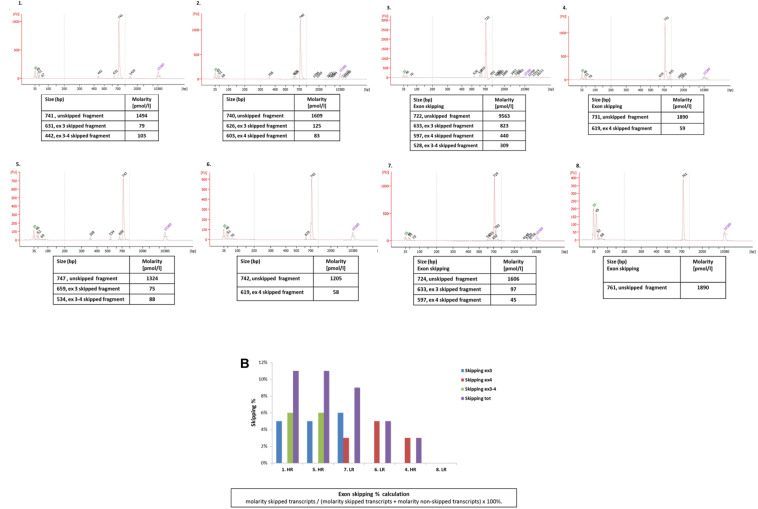
**(A)** The chromatograms show the size of the amplified fragments (exons 2–7) of TNFRSF10A genes of the 8 DMD patients listed in Table 1C. Concentrations (Molarity) related to the unskipped and skipped fragments are reported in the tables below the chromatograms; **(B)** the plot summarizes exon 3, exon 4, exons 3 and 4, and all exons skipping percentage. Patients are ordered in the graph based on their total skipping percentage higher values.

Figure 3 summarizes the results obtained by the Agilent high-sensitivity DNA chip, which shows the skipped and un-skipped fragments including the size and the concentration of the amplified fragments from the 8 DMD biopsies listed in Table 1C. The exon skipping percentages were calculated as described in section “Materials and Methods,” and the percentages of single exons (exon 3 and exon 4), both exons (exons 3–4) and total skipping amount are showed in Table 1C. The results revealed that the RNA analyzed from DMD biopsies showed skipping of exons 3, exon 4 and/or both in all patients, excluding DMD 8 (LR). The range of skipping percentage (3–7%) was low but well measurable using our assay. The higher levels of skipping were observed in DMD patients classified as HR. Among these HR patients, the 3 oldest DMD boys (gray label) showed the highest skipping percentages of both exons 3 and 4.

### TNFRSF10A ELISA Assay

The presence of TNFRSF10A in the blood stream is analyzed in plasma samples from DMD patients treated with deflazacort for at least 2 years. The CS treated patients are categorized as low responders, if the patients lost mobility before the age of 10, and high responders if the patients remained ambulant until the age of 15. Protein abundance was estimated in both serum and plasma as mean fluorescent intensities (MFI). The two antibodies targeting different epitopes of the TNFRSF10A protein have low signals in the two patient groups. In addition, Wilcoxon ranked-sum test showed no significant difference in abundance between high and low responders (Supplementary Figure S2). Although this result is preliminary, the possibility to dose TNFRSF10A receptor in plasma might be relevant for easy screening procedures in patients under CS treatment. Further validation studies are, however, needed to confirm the reliability of this method.

## Discussion

Although new therapeutic options have emerged in the last years for DMD boys (Scoto et al., 2018), CS treatment is considered the current standard of care for DMD in Europe, United States, Japan, and Australia (Bushby et al., 2010; Griggs et al., 2016). Nevertheless, because of the combination of variable response to their administration coupled with severe side effects, their monitoring would benefit from pharmacogenetics markers in order to predict drug response and to personalize the treatment.

Recent literature has experienced a flowering of interest in biomarkers in rare diseases, more specifically in DMD (Scotton et al., 2014; Ferlini et al., 2015; Vo and McNally, 2015; Szigyarto and Spitali, 2018). Both genetic modifiers influencing the disease course and biomarkers that might be used for therapy monitoring have been identified. The majority of these studies were focused on transcriptomic or proteomic signatures disclosing several biomarkers that are associated with disease severity or with specific disease signs, such as LoA, or disease signatures, such as muscle metabolism or regeneration (Hathout et al., 2016; Perry and Muntoni, 2016).

Only a few papers report on SNPs associated with DMD. Among these, SPP1 and LTBP4 are validated genetic modifiers linked to LoA in steroid-treated boys (Flanigan et al., 2013; Bello et al., 2015; van den Bergen et al., 2015), while promising new biomarkers, as ACTN3 and CD40, or THBS1 as locus modifier, were recently identified as associated with LoA in DMD boys (Bello et al., 2016; Hogarth et al., 2017; Weiss et al., 2018).

In order to identify biomarkers associated with CS response, we studied a small discovery cohort of 21 DMD patients (DiC) all treated by CS. We targeted 205 sequenced genes by NGS and selected 43 SNPs by statistical analysis. Among these SNPs, DAPC analysis prioritized two SNP genotypes, one in *TNFRSF10A* and the other in *VCAN*. Validation in three further DMD cohorts composed of 207 patients validated the CT alleles in the *TNFRSF10A* gene as candidate CS-responsiveness associated genotype.

### TNFRSF10A and Corticosteroid Response

*TNFRSF10A* was the top marker in the priority list based on DAPC analysis and resulted as strongly associated with high CS response (*P* < 0.005) but not with LoA in CS-untreated patients (*P* = 0.28).

TNFRSF10A, also called TRAIL-R1 or DR4, highly expressed in skeletal and cardiac muscle, is a member of the Tumor Necrosis Factor Receptor Superfamily or “death receptor family” and acts on the delicate balance between cell proliferation and death. It is indeed involved in inducing apoptosis but also in suppressing inflammation and metastasis (Zhang et al., 1999). Its ligand TRAIL (TNF-related apoptosis-inducing ligand) is known to promote cell proliferation and migration by activating the NF-kB pathway through its own receptor. TNFRSF10A also uses a different ligand, FADD-independent, to determine GR nucleus translocation (Dai et al., 2015).

Interestingly, the recently reported CD40 modifier acts on the same circuit (Bello et al., 2016). The *TNFRSF10A* SNPs identified in our analysis (rs17620, H141R, and rs20575, R209T) are missense variations predicted to be possibly damaging/benign by Polyphen (Supplementary Figure S1). They are also located within recognized exonic splicing enhancers in exon 3 and 4, respectively (both encoding part of the extracellular domain), possibly causing reduced ratio of exon 3 and/or 4 incorporation into the transcript (still being in frame, despite of the exon/exons omission). This is expected to cause modification of the TRAIL ligand domain composition which lies in the extracellular domain of TNFRSF10A, therefore reducing the TNF-related apoptotic effect. In order to determine the consequences on splicing to the identified SNPs we tested exons 3 and 4 skipping propensity and the TNFRSF10A transcript composition in 8 muscle biopsies from DMD with different haplotypes and different CS response (5 HR and 3 LR). Our RNA studies indeed showed that HR patients generally have a higher skipping propensity, with low but measurable skipping of exons 3, 4, or of both. Interestingly, the 3 more aged patients still ambulant have the highest skipping percentage and score of both exons. Conversely, patient 6, who is the heterozygous twin of patient 3, not ambulant and not carrying the C/T haplotype, does not have any exons 3–4 skipping and is a LR (Figure 3). This result suggests indeed that C/T haplotype may favor skipping of both exons 3 and 4 of the TNFRSF10A transcript. We speculate that the TNFRSF10A messenger skipping both exons will produce a shorter protein which misses part of the extracellular domain and therefore may reduce to some extent the detrimental, pro-apoptotic TRAIL-ligand effect, favoring a better CS response. Of course more patients should be studied to confirm this finding. The effect of rs17620 (H141R) is possibly damaging and has been associated with cancer risk (Dechant et al., 2004). We may speculate that these SNPs may modify the TRAIL binding and therefore its ability in triggering cells to die or to proliferate. This might play a balancing role in inducing DMD patients to be more or less responsive to CS therapy.

The *TNFRSF10A* missense variations might therefore modify CS response by keeping a fine balance between degeneration and regeneration, as reported by Fisher et al. in *mdx* mice (Fisher et al., 2005), thus reducing necrosis, as reported in cancer, by decreasing expression of death receptors (including TNFR), and eventually reducing apoptosis (Runnebaum and Brüning, 2005). Other important clues linking *TNFRSF10A* and CS are the liaison between CS and cytokine receptors and the cross-talk between TNF and GR signaling (Van Bogaert et al., 2010). Interestingly, and supporting the TNFR role in CS response, TNF family members were found to be downregulated in *mdx* mice treated with CS (Fisher et al., 2005).

**FIGURE 4:**
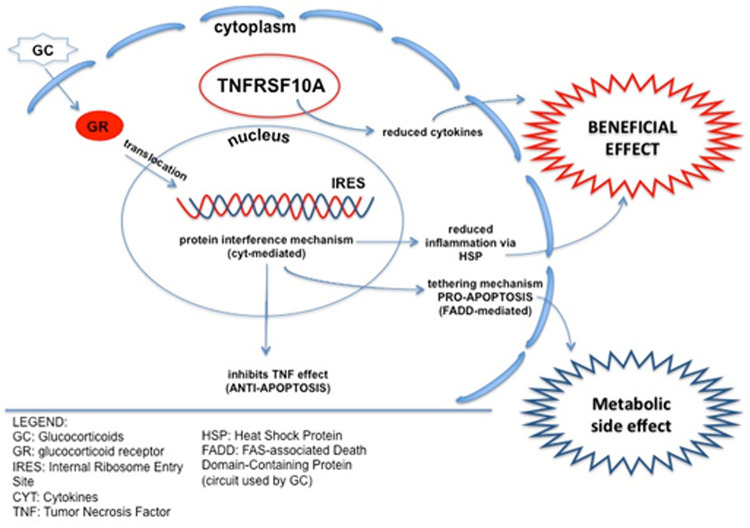
Schematic of crosstalk between the TNF-related pathway and CS response. TNFRSF10A is involved in inducing apoptosis, but also in suppressing inflammation, reducing pro-inflammation cytokines. As a possible underlining mechanism, TNFRSF10A can reduce the apoptotic effect of CS, via IRES elements, maintaining the anti-inflammatory action and potentially conferring a better CS response to HR patients. CS, Corticosteroids; GR, Glucocorticoid Receptor; IRES, Internal Ribosome Entry Site; CYT, Cytokines; TNF, Tumor Necrosis Factor; HSP, Heat Shock Protein; FADD, FAS-associated Death Domain-containing protein (circuit used by CS).

An example of such crosstalk is demonstration that TNFs potentiate the transactivation of GR, which protects the cell from the TNF-induced cytotoxicity (Van Bogaert et al., 2010). Therefore CS exerts a dual effect: (i) it induces apoptosis via activation of GR-IRES elements (cytokine mediated) but (ii) reduces TNF-induced apoptosis (see a model in Figure 4). It is worth mentioning that mutations in a different TNFR (*TNFRSF1A*) causes an autosomal dominant condition characterized by periodic fever and pain (OMIM #142680) which responds to CS but not to colchicine, fact that supports the TNFR role as CS modulator (Magnotti et al., 2013). Validation of *LTBP4* SNPs in our cohorts showed a predictive CS response role for genotypes but not for allelic distributions. These results might reflect the known predictive value for LTBP4 for LoA in DMD (Flanigan et al., 2013).

Taken together, these data suggest that *TNFRSF10A* may influence LoA in steroid users. It would be very interesting to test if the *TNFRSF10A* SNPs might be predictors of response of the recently described lazeroid steroidal backbone-based drug VBP15, a novel anti-inflammatory which improves muscle dystrophy without the CS negative effects (Heier et al., 2013; Sreetama et al., 2018).

## Conclusion

The identification of disease biomarkers or genetic modifiers in rare Mendelian diseases, as DMD, is very often complicated by the low number of patients with specific sub-phenotypes to be enrolled in clinical and genetic studies. Therefore, our results need further confirmation by studying larger DMD patient cohorts, via large validation studies. Here we suggest that the *TNFRSF10A* C/T haplotype confers a better response to CS since it reduces cytokines release and increases the beneficial effects of CS by decreasing their pro-apoptotic effect, TRAIL-mediated, likely via inducing exons 3 and 4 in frame skipping. A dual screening for TNFRSF10A and LTBP4 SNPs should be therefore carried out in DMD patients to explore the CS response. Our finding that TNFRSF10A is dosable in fluids by immunoassay may prompt studies aimed at testing haplotype/protein correlation in DMD patients.

Finally, we also highlight that TNFRSF family members are known therapeutic targets of enavatuzumab, which inhibits cancer growth and enhances the antitumor activity of several chemotherapy agents (Chao et al., 2013). Indeed, dystrophin has known oncosuppressor functions, although not completely explored, as recently pinpointed for its role in keeping genome stability via, at least in part, ROS release (Jelinkova et al., 2019) and its capacity to inhibit myogenic cell migration in sarcoma (Wang et al., 2014). Therefore, enavatuzumab, as well as possibly other molecules targeting TNF, might be further considered as possible therapeutics for DMD.

## Limitations of the Study

We would underline that our patients’ cohort is made of 217 DMD patients and it might therefore be suboptimal for statistical analysis. This is a well-known criticism for rare disease statistical studies, unfortunately unsolvable, since the low or even extremely low (as for the ultra-rare diseases) number of existing patients. In addition, when selecting a specific sub-phenotype, as corticosteroid response, loss of ambulation, cardiomyopathy, etc., the numbers become even more lower. This also applies to Duchenne muscular dystrophy. Therefore there is a large consensus about the utility of pilot studies (discovery) that can be performed on small patients’ numbers, followed by large validation studies, when feasible.

## Disclosure

AF is PI of Sarepta Therapeutics ongoing clinical trials for DMD, recipient of grants from PTC Therapeutics (DMD International) and Sarepta Therapeutics (limb girdle muscle dystrophies high throughput genetic diagnosis). AA-R discloses being employed by LUMC which has patents on exon skipping technology, some of which has been licensed to BioMarin and subsequently sublicensed to Sarepta. As co-inventor of some of these patents AA-R was entitled to a share of royalties. AA-R further discloses being *ad hoc* consultant for PTC Therapeutics, Sarepta Therapeutics, CRISPR Therapeutics, Summit PLC, Alpha Anomeric, BioMarin Pharmaceuticals Inc., Eisai, Global Guidepoint and GLG consultancy, Grunenthal, Wave and BioClinica, having been a member of the Duchenne Network Steering Committee (BioMarin) and being a member of the scientific advisory boards of ProQR and Philae Pharmaceuticals. Remuneration for these activities is paid to LUMC. LUMC also received speaker honoraria from PTC Therapeutics and BioMarin Pharmaceuticals and funding for contract research from Italpharmaco and Alpha Anomeric. FM has received grants and/or personal fees from Esperare, Pfizer, PTC Therapeutics, Santhera Pharmaceuticals, Sarepta Therapeutics, and Roche.

## Data Availability Statement

All datasets presented in this study are available as Supplementary Material. All SNPs identified and prioritized in this study are known and already present in public repositories (ExAC and dbSNP) and accession numbers can be found in the article and in Supplementary Tables S1–S4.

## Ethics Statement

The studies involving human participants were reviewed and approved by the S. Anna University Hospital Ferrara (Italy) Ethical Committee. Written informed consent to participate in this study was provided by the participants’ legal guardian/next of kin.

## Author Contributions

AF designed the rationale of the research, supervised the work, and wrote the manuscript. CP, RS, FD, RR, and MF performed the experimental procedures, data interpretation related to patients’ genetic and clinical details, and SNPs analyses. AC and CS designed and performed the statistical analyses and interpreted the data. PSa provided the muscle biopsy morphological data. KS and CA-K performed and interpreted the TNFRSF10A immunoassay on plasma and serum. MR, KB, VS, HL, FM, IZ, AD’A, EB, LM, MC, ST-G, SM, MP, EM, and FF performed the clinical assessment and provided patient samples. PB and GN revised the manuscript for the personalized medicine aspects. EK, ES, and ML performed the SNPs interactome analysis and data interpretation. RS, CS, PT’H, PSp, AA-R, ML, and FM critically revised the manuscript.

## Conflict of Interest

EK and ML were employed by the company Panacea Pharmaceuticals, United States. The remaining authors declare that the research was conducted in the absence of any commercial or financial relationships that could be construed as a potential conflict of interest.

## Footnotes

1 https://www.fda.gov/NewsEvents/Newsroom/PressAnnouncements/ucm540945.htm

2 https://www.ucl.ac.uk/child-health/mrc-cnmd-biobank-london

3 www.bio-nmd.eu

4 www.lifetechnologies.com/lifescope

5 http://exac.broadinstitute.org

6 http://www.funrich.org

## Supplementary Material

FIGURE S1 TNFRS10A gene structure **(A)**, SNPs annotation and gene position **(B)**, and interactome analysis **(C)**.

FIGURE S2 Antibody-based proteomics analysis in blood samples. Abundance of TNSFRF10A was estimated using 2 antibodies HPA054475 **(A)** and anti-TNFSRF10A **(B)** in plasma samples collected from patients treated with corticosteroids.

TABLE S1 List of the prioritized DMD-associated genes.

TABLE S2 **(A)** List of primers for SNP validation; **(B)** allelic and genotypic frequencies of SPP1 and LTBP4 and statistical results.

TABLE S3 SNPs selected after the SOLiD sequencing enrichment.

TABLE S4 DAPC analysis SNP output.
