# Supplementary material for: Tumor Necrosis Factor Receptor SF10A (TNFRSF10A) SNPs Correlate With Corticosteroid Response in Duchenne Muscular Dystrophy
Source: Front Genet. 2020 Jul 3;11:605. doi: 10.3389/fgene.2020.00605 (PMC7350910; doi:10.3389/fgene.2020.00605)

Figure S1.

A) Scheme of TNFRSF10A gene and protein. The gene consists of 10 exons, which codify for 4 main functional domains. The SNPs identified by our analysis are localized in the extracellular domain.

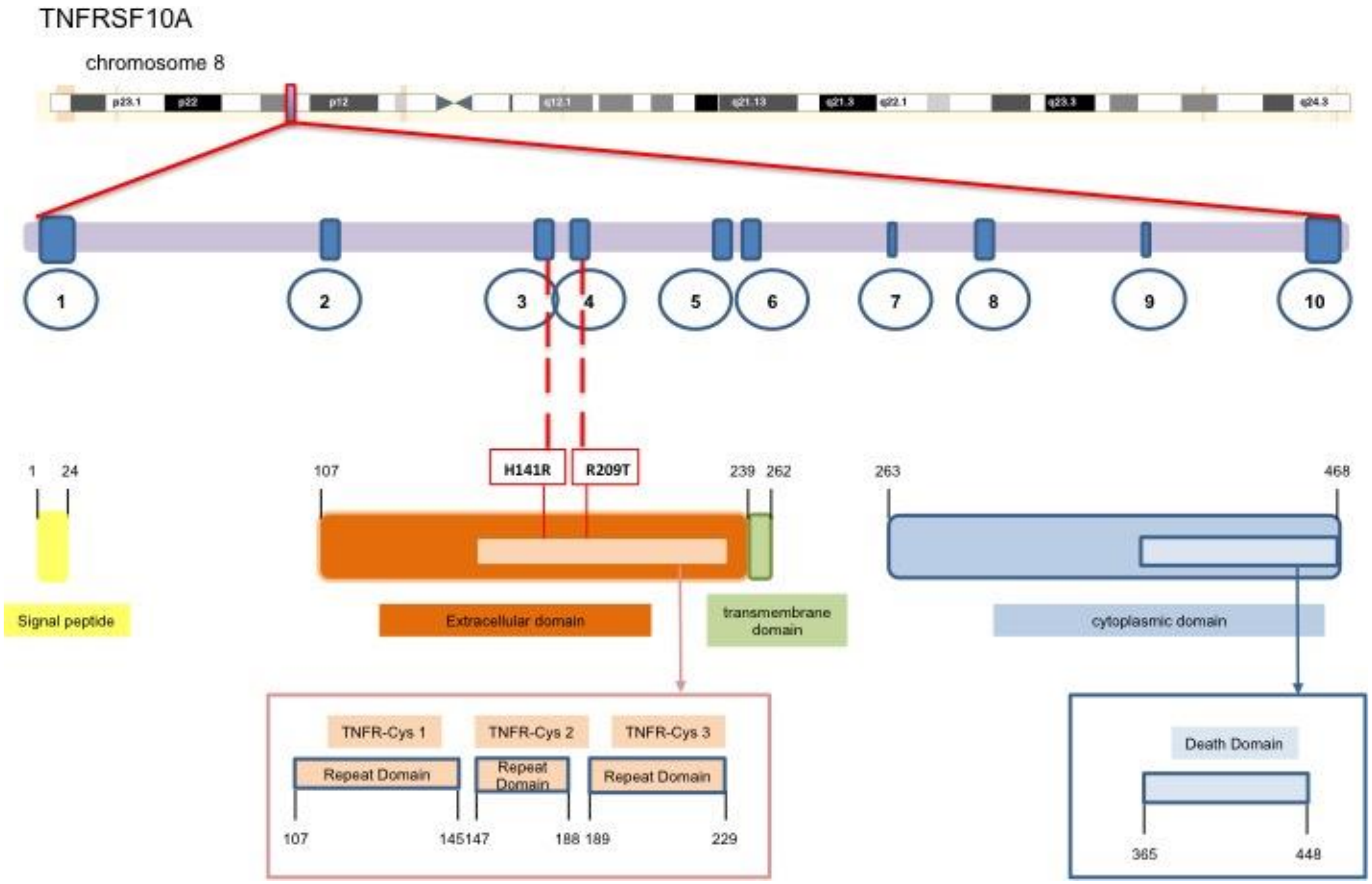

B)

snp	gene	Cr.	reference	exon	nucleotide change	alleles frequency		protein change	functional effect	Polyphen prediction
**rs20575**	TNFRSF10A	8	NM_003844	4/10	c.626G>C	50%	50%	p.Arg209Thr	missense	benign
**rs6557634 (it has merged into rs17620)**	TNFRSF10A	8	NM_003844	3/10	c.422A>G	65%	35%	p.His141Arg	missense	Possibly damage

C) Interactome of TNFRS10A protein built up using SPRING (Pathway Studio software)

HIF1A-TNFRSF10A-NFE2L2 pathway

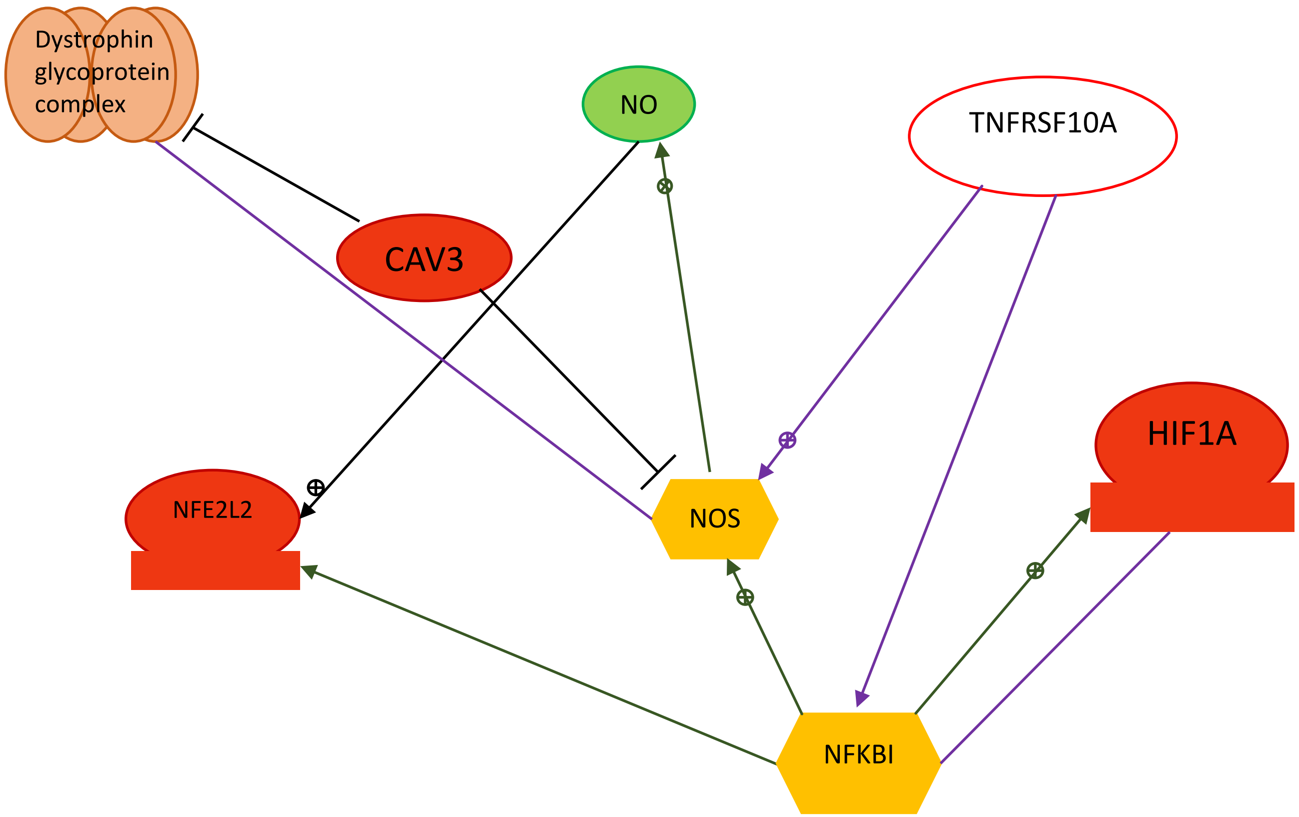

Supplement: FIGURE S1 — TNFRS10A gene structure (A), SNPs annotation and gene position (B), and interactome analysis (C). [file Image_1.pdf]
