# Supplementary material for: Tumor Necrosis Factor Receptor SF10A (TNFRSF10A) SNPs Correlate With Corticosteroid Response in Duchenne Muscular Dystrophy
Source: Front Genet. 2020 Jul 3;11:605. doi: 10.3389/fgene.2020.00605 (PMC7350910; doi:10.3389/fgene.2020.00605)
Supplement: TABLE S1 — List of the prioritized DMD-associated genes. [file Table_1.docx]

**Table S1. List of the DMD-associated genes.** List of the top 205 genes prioritized from the BIO-NMD project and selected for the SOLiD sequencing enrichment. Different Refseq of the same gene have been reported.

| number | Refseq SNP | Gene |
| --- | --- | --- |
| 1 | NM_005026 | PIK3CD |
| 2 | NM_014654 | SDC3 |
| 3 | NM_001142588 | NFYC |
| 4 | NM_001114172 | PIK3R3 |
| 5 | NM_006252 | PRKAA2 |
| 6 | NM_002228 | JUN |
| 7 | NM_172212 | CSF1 |
| 8 | NM_003637 | ITGA10 |
| 9 | NM_005399 | PRKAB2 |
| 10 | NM_181715 | CRTC2 |
| 11 | NM_002293 | LAMC1 |
| 12 | NM_005562 | LAMC2 |
| 13 | NM_133262, NM_133326 | ATP6V1G3 |
| 14 | NM_002479 | MYOG |
| 15 | NM_002646 | PIK3C2B |
| 16 | NM_001017402 | LAMB3 |
| 17 | NM_001040619, NM_001030287 | ATF3 |
| 18 | NM_001134285 | ESRRG |
| 19 | NM_002189 | IL15RA |
| 20 | NM_003638 | ITGA8 |
| 21 | NM_033668 | ITGB1 |
| 22 | NM_012238 | SIRT1 |
| 23 | NM_003373, NM_014000 | VCL |
| 24 | NM_003375 | VDAC2 |
| 25 | NM_002645 | PIK3C2A |
| 26 | NM_000614 | CNTF |
| 27 | NM_004451 | ESRRA |
| 28 | NM_138456 | BATF2 |
| 29 | NM_005438 | FOSL1 |
| 30 | NM_001104 | ACTN3 |
| 31 | NM_001143836 | NOX4 |
| 32 | NM_000552 | VWF |
| 33 | NM_004570 | PIK3C2G |
| 34 | NM_005086, NM_001135823 | SSPN |
| 35 | NM_001206709 | PRKAG1 |
| 36 | NM_000889 | ITGB7 |
| 37 | NM_002205 | ITGA5 |
| 38 | NM_002206, NM_001144997 | ITGA7 |
| 39 | NM_002392 | MDM2 |
| 40 | NM_133503 | DCN |
| 41 | NM_006166 | NFYB |
| 42 | NM_006253 | PRKAB1 |
| 43 | NM_025157, NM_001080855 | PXN |
| 44 | NM_000231 | SGCG |
| 45 | NM_004791 | ITGBL1 |
| 46 | NM_001845 | COL4A1 |
| 47 | NM_001846 | COL4A2 |
| 48 | NM_002471 | MYH6 |
| 49 | NM_000257 | MYH7 |
| 50 | NM_005252 | FOS |
| 51 | NM_001135049, NM_001135047 | JDP2 |
| 52 | NM_006399 | BATF |
| 53 | NM_001004439 | ITGA11 |
| 54 | NM_024505, NM_001184780 | NOX5 |
| 55 | NM_000246 | CIITA |
| 56 | NM_015092 | SMG1 |
| 57 | NM_001040056 | MAPK3 |
| 58 | NM_002209 | ITGAL |
| 59 | NM_000632, NM_001145808 | ITGAM |
| 60 | NM_000887 | ITGAX |
| 61 | NM_005353 | ITGAD |
| 62 | NM_001127891 | MMP2 |
| 63 | NM_020313 | CIAPIN1 |
| 64 | NM_004691 | ATP6V0D1 |
| 65 | NM_006750 | SNTB2 |
| 66 | NM_001170720, NM_001170714 | BCAR1 |
| 67 | NM_002208 | ITGAE |
| 68 | NM_001142633 | PIK3R5 |
| 69 | NM_003802 | MYH13 |
| 70 | NM_002472 | MYH8 |
| 71 | NM_017533 | MYH4 |
| 72 | NM_005963 | MYH1 |
| 73 | NM_017534 | MYH2 |
| 74 | NM_002470 | MYH3 |
| 75 | NM_001005291 | SREBF1 |
| 76 | NM_000638 | VTN |
| 77 | NM_002982 | CCL2 |
| 78 | NM_003250 | THRA |
| 79 | NM_005177 | ATP6V0A1 |
| 80 | NM_013999 | MEOX1 |
| 81 | NM_000419 | ITGA2B |
| 82 | NM_000212 | ITGB3 |
| 83 | NM_005501 | ITGA3 |
| 84 | NM_000023 | SGCA |
| 85 | NM_000088 | COL1A1 |
| 86 | NM_000789, NM_001178057 | ACE |
| 87 | NM_000515 | GH1 |
| 88 | NM_001005619 | ITGB4 |
| 89 | NM_001614 | ACTG1 |
| 90 | NM_005559 | LAMA1 |
| 91 | NM_005406 | ROCK1 |
| 92 | NM_001127717, NM_001127718 | LAMA3 |
| 93 | NM_001198939, NM_032981 | DTNA |
| 94 | NM_002647 | PIK3C3 |
| 95 | NM_001190821, NM_001190823 | SMAD7 |
| 96 | NM_000657 | BCL2 |
| 97 | NM_005027 | PIK3R2 |
| 98 | NM_002911 | UPF1 |
| 99 | NM_002503 | NFKBIB |
| 100 | NM_001042544 | LTBP4 |
| 101 | NM_000660 | TGFB1 |
| 102 | NM_007121 | NR1H2 |
| 103 | NM_001039362 | ATP6V1C2 |
| 104 | NM_001006946 | SDC1 |
| 105 | NM_033147 | DTNB |
| 106 | NM_005253 | FOSL2 |
| 107 | NM_001692 | ATP6V1B1 |
| 108 | NM_000575 | IL1A |
| 109 | NM_000576 | IL1B |
| 110 | NM_014440 | IL36A |
| 111 | NM_014438, NM_173178 | IL36B |
| 112 | NM_173842, NM_000577 | IL1RN |
| 113 | NM_000888 | ITGB6 |
| 114 | NM_001935 | DPP4 |
| 115 | NM_002054 | GCG |
| 116 | NM_000210, NM_001079818 | ITGA6 |
| 117 | NM_000885 | ITGA4 |
| 118 | NM_001145000 | ITGAV |
| 119 | NM_005259 | MSTN |
| 120 | NM_001608 | ACADL |
| 121 | NM_004044 | ATIC |
| 122 | NM_212482 | FN1 |
| 123 | NM_017431 | PRKAG3 |
| 124 | NM_000092 | COL4A4 |
| 125 | NM_000091 | COL4A3 |
| 126 | NM_057166, NM_057164, NM_057167 | COL6A3 |
| 127 | NM_001200 | BMP2 |
| 128 | NM_003098 | SNTA1 |
| 129 | NM_005560 | LAMA5 |
| 130 | NM_001853 | COL9A3 |
| 131 | NM_001697 | ATP5O |
| 132 | NM_001001890 | RUNX1 |
| 133 | NM_000211 | ITGB2 |
| 134 | NM_001848 | COL6A1 |
| 135 | NM_058174, NM_001849 | COL6A2 |
| 136 | NM_001039366 | ATP6V1E1 |
| 137 | NM_001196 | BID |
| 138 | NM_138957 | MAPK1 |
| 139 | NM_004599 | SREBF2 |
| 140 | NM_001001928 | PPARA |
| 141 | NM_002880 | RAF1 |
| 142 | NM_002207 | ITGA9 |
| 143 | NM_001123041, NM_001123396 | CCR2 |
| 144 | NM_002292 | LAMB2 |
| 145 | NM_001177636 | DAG1 |
| 146 | NM_001690 | ATP6V1A |
| 147 | NM_001178065 | CASR |
| 148 | NM_002213 | ITGB5 |
| 149 | NM_014602 | PIK3R4 |
| 150 | NM_006219 | PIK3CB |
| 151 | NM_006218 | PIK3CA |
| 152 | NM_001177800 | ADIPOQ |
| 153 | NM_001130845 | BCL6 |
| 154 | NM_013261 | PPARGC1A |
| 155 | NM_005038 | PPID |
| 156 | NM_032991 | CASP3 |
| 157 | NM_001151 | SLC25A4 |
| 158 | NM_181501 | ITGA1 |
| 159 | NM_002203 | ITGA2 |
| 160 | NM_181523, NM_181504, NM_001242466 | PIK3R1 |
| 161 | NM_001126336, NM_001164098, NM_001164097 | VCAN |
| 162 | NM_002188 | IL13 |
| 163 | NM_000589 | IL4 |
| 164 | NM_003374 | VDAC1 |
| 165 | NM_001018074 | NR3C1 |
| 166 | NM_172244 | SGCD |
| 167 | NM_001171819 | PPARD |
| 168 | NM_001105207, NM_001105208 | LAMA4 |
| 169 | NM_001079823 | LAMA2 |
| 170 | NM_001122740 | ESR1 |
| 171 | NM_015718 | NOX3 |
| 172 | NM_001101 | ACTB |
| 173 | NM_002214 | ITGB8 |
| 174 | NM_000600 | IL6 |
| 175 | NM_000089 | COL1A2 |
| 176 | NM_005746 | NAMPT |
| 177 | NM_002649 | PIK3CG |
| 178 | NM_002291 | LAMB1 |
| 179 | NM_007356 | LAMB4 |
| 180 | NM_000245 | MET |
| 181 | NM_000230 | LEP |
| 182 | NM_001628 | AKR1B1 |
| 183 | NM_130840 | ATP6V0A4 |
| 184 | NM_001040633, NM_016203 | PRKAG2 |
| 185 | NM_139167 | SGCZ |
| 186 | NM_001693 | ATP6V1B2 |
| 187 | NM_003844 | TNFRSF10A |
| 188 | NM_005662 | VDAC3 |
| 189 | NM_015941 | ATP6V1H |
| 190 | NM_152565 | ATP6V0D2 |
| 191 | NM_004349 | RUNX1T1 |
| 192 | NM_002998 | SDC2 |
| 193 | NM_001695 | ATP6V1C1 |
| 194 | NM_001199649, NM_005607 | PTK2 |
| 195 | NM_001916 | CYC1 |
| 196 | NM_001127610 | BAAT |
| 197 | NM_001099679 | TRIM32 |
| 198 | NM_006059 | LAMC3 |
| 199 | NM_002957 | RXRA |
| 200 | NM_004016, NM_004007, NM_000109 | DMD |
| 201 | NM_001170931 | FOXO4 |
| 202 | NM_000291 | PGK1 |
| 203 | NM_007052 | NOX1 |
| 204 | NM_033641 | COL4A6 |
| 205 | NM_000495 | COL4A5 |
