# Supplementary material for: Tumor Necrosis Factor Receptor SF10A (TNFRSF10A) SNPs Correlate With Corticosteroid Response in Duchenne Muscular Dystrophy
Source: Front Genet. 2020 Jul 3;11:605. doi: 10.3389/fgene.2020.00605 (PMC7350910; doi:10.3389/fgene.2020.00605)
Supplement: TABLE S2 — (A) List of primers for SNP validation; (B) allelic and genotypic frequencies of SPP1 and LTBP4 and statistical results. [file Table_2.doc]

**Table S2.**

1. **Pairs of primers used for validation analysis.** All the DMD patients in the Validation Cohort (VaC) were genotyped for the *TNFRSF10A, VCAN, LTBP4, GLCCI2, CRHR1 and SPP1* SNPs.

| **GENE** | **EXON** | **Primer Forward** | **Primer Reverse** | **Amplification**  **fragment (bp)** | **SNP** | **detected variation** |
| --- | --- | --- | --- | --- | --- | --- |
| TNFRSF10A | 4 | ctggccctcagaactccttg | aggttgcaggctcaggaga | 407 | rs 20575 | c.626G>C  p.R209T |
| TNFRSF10A | 3 | ggtgtgtatagatgcagaattgacg | tggaacagggtatgatgaagacc | 398 | rs17620 | c.422A>G  p.H141R |
| VCAN | 4 | ttggacgttggggcagtcat | tgaacttctggtcttccctcagt | 337 | rs 4470745 | c.645A>G  p.V215= |
| VCAN | 2 | tgccacccagttacaacacca | tccgctcatccaaaccccaa | 387 | rs 12332199 | c.348T>C  p.Thr116= |
| LTBP4 | 5 | ggccacatgacagctaaggg | gctcgggaaggcgggaaata | 399 | rs2303729 | c.580G>A  p.V194I |
| LTBP4 | 17 | cagaaggcaggctcaagact | aggaggttacggccaggt | 525 | rs 1131620  rs1051303 | c.2359A>G  p.T787A  c.2458A>G  p.T820A |
| LTBP4 | 26 | agaggagcgtgagaggtgtg | accccacccagtatttccacc | 478 | rs10880 | c.3422C>T  p.T1141M |
| GLCCI2 |  | aagctatctgcttcggaaaagc | tgggccgtctgtatgacttg | 378 | rs37973 | c.-1106G>A |
| CRHR1 |  | acacctgcagccgacctt | ctggcttccagcctggtg | 316 | rs1876828 | c.1107+111C>T |
| SPP1 |  | aagtgctcttcctggatgctg | ctcctgctgctgctgacaac | 275 | rs28357094 | c.-170T>G |

1. **Allelic and genotypic frequencies of SPP1 and LTBP4 genes in Low Responders (LR) and High Responders (HR) tested in DiC and VaC1 cohorts; RefSNP Alleles (A1, A2 where A1 is the ancestral allele); contingency χ2 (Pearson Uncorrected) and G test probability value (P).**

| Gene | dbSNP code | A1 | A2 | TEST | LR | HR | DF | χ^2^ | P | G test P |
| --- | --- | --- | --- | --- | --- | --- | --- | --- | --- | --- |
| SPP1 | rs28357094 | G | T | GENOTYPIC | 0/2/9 | 0/8/11 | 1 | 1.79 | 0.180 | 0.168 |
|  |  | G | T | ALLELIC | 2/20 | 8/30 | 1 | 1.44 | 0.231 |  |
| LTBP4 | rs2303729 | A | G | GENOTYPIC | 2/3/6 | 5/12/2 | 2 | 7.05 | 0.029 | 0.030 |
|  |  | A | G | ALLELIC | 7/15 | 22/16 | 1 | 3.79 | 0.051 |  |
|  | rs1131620 | G | A | GENOTYPIC | 2/3/6 | 4/13/2 | 2 | 7.30 | 0.026 | 0.025 |
|  |  | G | A | ALLELIC | 7/15 | 21/17 | 1 | 3.08 | 0.079 |  |
|  | rs1051303 | G | A | GENOTYPIC | 2/3/6 | 4/13/2 | 2 | 7.30 | 0.026 | 0.025 |
|  |  | G | A | ALLELIC | 7/15 | 21/17 | 1 | 3.08 | 0.079 |  |
|  | rs10880 | T | C | GENOTYPIC | 1/3/7 | 3/12/4 | 2 | 5.47 | 0.650 | 0.064 |
|  |  | T | C | ALLELIC | 5/17 | 18/20 | 1 | 3.58 | 0.059 |  |
