# Supplementary material for: Tumor Necrosis Factor Receptor SF10A (TNFRSF10A) SNPs Correlate With Corticosteroid Response in Duchenne Muscular Dystrophy
Source: Front Genet. 2020 Jul 3;11:605. doi: 10.3389/fgene.2020.00605 (PMC7350910; doi:10.3389/fgene.2020.00605)
Supplement: TABLE S3 — SNPs selected after the SOLiD sequencing enrichment. [file Table_3.doc]

**Table S3. SNPs selected after the SOLiD sequencing enrichment.** The 354 SNPs have been prioritized considering a medium/high allele frequency in the dbSNP database and on the basis of quality filters. In yellow and green SNPs in the same genes.

| Number | GENE | Refseq | SNP |
| --- | --- | --- | --- |
| 1 | PIK3CD | NM_005026 | rs11121484 |
| 2 | SDC3 | NM_014654 | rs4949184 |
| 3 | NFYC | NM_001142588 | rs1057925 |
| 4 | PIK3R3 | NM_001114172 | rs785468 |
| 5 | PIK3R3 | NM_001114172 | rs785467 |
| 6 | PRKAA2 | NM_006252 | rs55717918 |
| 7 | ITGA10 | NM_003637 | rs11590105 |
| 8 | ITGA10 | NM_003637 | rs2274616 |
| 9 | LAMC1 | NM_002293 | rs2296288 |
| 10 | LAMC1 | NM_002293 | rs2296289 |
| 11 | LAMC1 | NM_002293 | rs20563 |
| 12 | LAMC1 | NM_002293 | rs2296292 |
| 13 | LAMC1 | NM_002293 | rs20557 |
| 14 | LAMC1 | NM_002293 | rs20558 |
| 15 | LAMC1 | NM_002293 | rs61749262 |
| 16 | LAMC1 | NM_002293 | rs20560 |
| 17 | LAMC2 | NM_005562 | rs2274980 |
| 18 | LAMC2 | NM_005562 | rs11586699 |
| 19 | LAMC2 | NM_005562 | rs1129723 |
| 20 | LAMC2 | NM_005562 | rs2296306 |
| 21 | LAMC2 | NM_005562 | rs1047980 |
| 22 | LAMC2 | NM_005562 | rs2296303 |
| 23 | LAMC2 | NM_005562 | rs1047981 |
| 24 | PIK3C2B | NM_002646 | rs17334387 |
| 25 | PIK3C2B | NM_002646 | rs3747636 |
| 26 | PIK3C2B | NM_002646 | rs1124777 |
| 27 | PIK3C2B | NM_002646 | rs1553921 |
| 28 | LAMB3 | NM_001017402 | rs2076356 |
| 29 | LAMB3 | NM_001017402 | rs3764781 |
| 30 | ESRRG | NM_001134285 | rs945453 |
| 31 | ATP6V1C2 | NM_001039362 | rs1198849 |
| 32 | ATP6V1C2 | NM_001039362 | rs72777388 |
| 33 | IL1A | NM_000575 | rs17561 |
| 34 | IL1B | NM_000576 | rs1143634 |
| 35 | IL36A | NM_014440 | rs895497 |
| 36 | IL1RN | NM_000577 | rs419598 |
| 37 | IL1RN | NM_000577 | rs315952 |
| 38 | ITGB6 | NM_000888 | rs16844790 |
| 39 | ITGB6 | NM_000888 | rs2305819 |
| 40 | ITGB6 | NM_000888 | rs2305818 |
| 41 | DPP4 | NM_001935 | rs41268649 |
| 42 | DPP4 | NM_001935 | rs17574 |
| 43 | ITGA6 | NM_000210 | rs11895564 |
| 44 | ITGA6 | NM_000210 | rs2293649 |
| 45 | ITGA6 | NM_000210 | rs2293648 |
| 46 | ITGA4 | NM_000885 | rs1143676 |
| 47 | ATIC | NM_004044 | rs2372536 |
| 48 | FN1 | NM_212482 | rs11651 |
| 49 | FN1 | NM_212482 | rs1132741 |
| 50 | FN1 | NM_212482 | rs13652 |
| 51 | FN1 | NM_212482 | rs1053238 |
| 52 | FN1 | NM_212482 | rs7589580 |
| 53 | FN1 | NM_212482 | rs7596677 |
| 54 | FN1 | NM_212482 | rs2289202 |
| 55 | COL4A4 | NM_000092 | rs2228557 |
| 56 | COL4A4 | NM_000092 | rs3817490 |
| 57 | COL4A4 | NM_000092 | rs2229813 |
| 58 | COL4A4 | NM_000092 | rs2229812 |
| 59 | COL4A4 | NM_000092 | rs10203363 |
| 60 | COL4A4 | NM_000092 | rs1800517 |
| 61 | COL4A4 | NM_000092 | rs2229814 |
| 62 | COL4A3 | NM_000091 | rs13424243 |
| 63 | COL4A3 | NM_000091 | rs10178458 |
| 64 | COL4A3 | NM_000091 | rs6436669 |
| 65 | COL4A3 | NM_000091 | rs55703767 |
| 66 | COL4A3 | NM_000091 | rs28381984 |
| 67 | COL6A3 | NM_057166 | rs112290343 |
| 68 | COL6A3 | NM_057166 | rs2270671 |
| 69 | COL6A3 | NM_057166 | rs1131296 |
| 70 | COL6A3 | NM_057166 | rs2270669 |
| 71 | COL6A3 | NM_057166 | rs61729844 |
| 72 | COL6A3 | NM_057166 | rs4433949 |
| 73 | COL6A3 | NM_057166 | rs34558385 |
| 74 | COL6A3 | NM_057166 | rs2291795 |
| 75 | COL6A3 | NM_057166 | rs2646258 |
| 76 | COL6A3 | NM_057166 | rs34181055 |
| 77 | COL6A3 | NM_057166 | rs2646265 |
| 78 | COL6A3 | NM_057166 | rs36117715 |
| 79 | COL6A3 | NM_057166 | rs2646254 |
| 80 | COL6A3 | NM_057166 | rs61729839 |
| 81 | COL6A3 | NM_057166 | rs2645774 |
| 82 | COL6A3 | NM_057166 | rs2646260 |
| 83 | COL6A3 | NM_057166 | rs12622093 |
| 84 | ITGA9 | NM_002207 | rs17227748 |
| 85 | ITGA9 | NM_002207 | rs267561 |
| 86 | CCR2 | NM_001123041 | rs1799864 |
| 87 | CCR2 | NM_001123041 | rs1799865 |
| 88 | LAMB2 | NM_002292 | rs34290943 |
| 89 | LAMB2 | NM_002292 | rs79448908 |
| 90 | DAG1 | NM_001177636 | rs1801143 |
| 91 | CASR | NM_001178065 | rs1042636 |
| 92 | CASR | NM_001178065 | rs1801726 |
| 93 | ITGB5 | NM_002213 | rs1803825 |
| 94 | PIK3R4 | NM_014602 | rs2170990 |
| 95 | PIK3CB | NM_006219 | rs3729780 |
| 96 | PPARGC1A | NM_013261 | rs3755863 |
| 97 | PPARGC1A | NM_013261 | rs8192678 |
| 98 | PPARGC1A | NM_013261 | rs17574213 |
| 99 | PPARGC1A | NM_013261 | rs2970847 |
| 100 | PPID | NM_005038 | rs9410 |
| 101 | PPID | NM_005038 | rs2230221 |
| 102 | PPID | NM_005038 | rs2070631 |
| 103 | ITGA1 | NM_181501 | rs2447867 |
| 104 | ITGA1 | NM_181501 | rs1531545 |
| 105 | ITGA1 | NM_181501 | rs4145748 |
| 106 | ITGA1 | NM_181501 | rs2279587 |
| 107 | ITGA1 | NM_181501 | rs12520591 |
| 108 | ITGA2 | NM_002203 | rs1126643 |
| 109 | ITGA2 | NM_002203 | rs1139484 |
| 110 | ITGA2 | NM_002203 | rs1062535 |
| 111 | ITGA2 | NM_002203 | rs3212523 |
| 112 | ITGA2 | NM_002203 | rs3212583 |
| 113 | ITGA2 | NM_002203 | rs3212327 |
| 114 | ITGA2 | NM_002203 | rs3213805 |
| 115 | ITGA2 | NM_002203 | rs80331976 |
| 116 | ITGA2 | NM_002203 | rs2303122 |
| 117 | PIK3R1 | NM_181523 | rs706713 |
| 118 | PIK3R1 | NM_181504 | rs3730089 |
| 119 | VCAN | NM_001126336 | rs12332199 |
| 120 | VCAN | NM_001126336 | rs4470745 |
| 121 | VCAN | NM_001164098 | rs2287926 |
| 122 | VCAN | NM_001164097 | rs2548541 |
| 123 | VCAN | NM_001164097 | rs309559 |
| 124 | VCAN | NM_001164097 | rs16900528 |
| 125 | VCAN | NM_001164097 | rs188703 |
| 126 | VCAN | NM_001164097 | rs309557 |
| 127 | VCAN | NM_001164097 | rs160279 |
| 128 | VCAN | NM_001164097 | rs160278 |
| 129 | VCAN | NM_001164097 | rs61754536 |
| 130 | VCAN | NM_001164097 | rs160277 |
| 131 | VCAN | NM_001126336 | rs308365 |
| 132 | IL13 | NM_002188 | rs20541 |
| 133 | NR3C1 | NM_001018074 | rs6196 |
| 134 | NR3C1 | NM_001018074 | rs56149945 |
| 135 | SGCD | NM_172244 | rs1801193 |
| 136 | LAMA4 | NM_001105207 | rs1050353 |
| 137 | LAMA4 | NM_001105207 | rs1050349 |
| 138 | LAMA4 | NM_001105207 | rs2032567 |
| 139 | LAMA4 | NM_001105207 | rs41289902 |
| 140 | LAMA4 | NM_001105207 | rs3752577 |
| 141 | LAMA4 | NM_001105207 | rs1050348 |
| 142 | LAMA4 | NM_001105207 | rs2072021 |
| 143 | LAMA4 | NM_001105207 | rs11757455 |
| 144 | LAMA2 | NM_001079823 | rs1140366 |
| 145 | LAMA2 | NM_001079823 | rs4404787 |
| 146 | LAMA2 | NM_001079823 | rs1027199 |
| 147 | LAMA2 | NM_001079823 | rs2306942 |
| 148 | LAMA2 | NM_001079823 | rs35089085 |
| 149 | LAMA2 | NM_001079823 | rs117781224 |
| 150 | LAMA2 | NM_001079823 | rs17057184 |
| 151 | LAMA2 | NM_001079823 | rs3749877 |
| 152 | LAMA2 | NM_001079823 | rs3749878 |
| 153 | LAMA2 | NM_001079823 | rs2297738 |
| 154 | LAMA2 | NM_001079823 | rs6569605 |
| 155 | LAMA2 | NM_001079823 | rs6569606 |
| 156 | LAMA2 | NM_001079823 | rs2229850 |
| 157 | ESR1 | NM_001122740 | rs1801132 |
| 158 | NOX3 | NM_015718 | rs231954 |
| 159 | NOX3 | NM_015718 | rs3749930 |
| 160 | NOX3 | NM_015718 | rs34960420 |
| 161 | NOX3 | NM_015718 | rs12195525 |
| 162 | ITGB8 | NM_002214 | rs3735619 |
| 163 | ITGB8 | NM_002214 | rs6968952 |
| 164 | COL1A2 | NM_000089 | rs1800222 |
| 165 | COL1A2 | NM_000089 | rs42524 |
| 166 | COL1A2 | NM_000089 | rs1800248 |
| 167 | PIK3CG | NM_002649 | rs17847825 |
| 168 | PIK3CG | NM_002649 | rs1129293 |
| 169 | LAMB1 | NM_002291 | rs1131398 |
| 170 | LAMB1 | NM_002291 | rs20556 |
| 171 | LAMB1 | NM_002291 | rs35710474 |
| 172 | LAMB1 | NM_002291 | rs2230156 |
| 173 | LAMB1 | NM_002291 | rs11770141 |
| 174 | LAMB1 | NM_002291 | rs25659 |
| 175 | LAMB4 | NM_007356 | rs2528693 |
| 176 | LAMB4 | NM_007356 | rs1627354 |
| 177 | LAMB4 | NM_007356 | rs9690688 |
| 178 | LAMB4 | NM_007356 | rs451252 |
| 179 | MET | NM_000245 | rs11762213 |
| 180 | MET | NM_000245 | rs55985569 |
| 181 | MET | NM_000245 | rs13223756 |
| 182 | MET | NM_000245 | rs41736 |
| 183 | MET | NM_000245 | rs2023748 |
| 184 | MET | NM_000245 | rs41737 |
| 185 | ATP6V0A4 | NM_130840 | rs3807154 |
| 186 | ATP6V0A4 | NM_130840 | rs1026435 |
| 187 | ATP6V0A4 | NM_130840 | rs74921348 |
| 188 | ATP6V0A4 | NM_130840 | rs10258719 |
| 189 | TNFRSF10A | NM_003844 | rs2230229 |
| 190 | TNFRSF10A | NM_003844 | rs20576 |
| 191 | TNFRSF10A | NM_003844 | rs20575 |
| 192 | TNFRSF10A | NM_003844 | rs6557634 |
| 193 | SDC2 | NM_002998 | rs1126681 |
| 194 | SDC2 | NM_002998 | rs1042381 |
| 195 | BAAT | NM_001127610 | rs41281027 |
| 196 | BAAT | NM_001127610 | rs1572983 |
| 197 | TRIM32 | NM_001099679 | rs1661300 |
| 198 | IL15RA | NM_002189 | rs2228059 |
| 199 | IL15RA | NM_002189 | rs2296139 |
| 200 | ITGA8 | NM_003638 | rs9333269 |
| 201 | ITGB1 | NM_033668 | rs2230396 |
| 202 | ITGB1 | NM_033668 | rs2230395 |
| 203 | ITGB1 | NM_033668 | rs2298141 |
| 204 | ITGB1 | NM_033668 | rs2230394 |
| 205 | SIRT1 | NM_012238 | rs2273773 |
| 206 | VCL | NM_003373 | rs767809 |
| 207 | VCL | NM_014000 | rs2131956 |
| 208 | PIK3C2A | NM_002645 | rs11604561 |
| 209 | PIK3C2A | NM_002645 | rs214936 |
| 210 | ACTN3 | NM_001104 | rs77239910 |
| 211 | ACTN3 | NM_001104 | rs540874 |
| 212 | NOX4 | NM_001143836 | rs317139 |
| 213 | VWF | NM_000552 | rs7962217 |
| 214 | VWF | NM_000552 | rs216902 |
| 215 | VWF | NM_000552 | rs1800380 |
| 216 | VWF | NM_000552 | rs1063857 |
| 217 | VWF | NM_000552 | rs1063856 |
| 218 | VWF | NM_000552 | rs1800378 |
| 219 | VWF | NM_000552 | rs1800376 |
| 220 | VWF | NM_000552 | rs1800375 |
| 221 | PIK3C2G | NM_004570 | rs11044004 |
| 222 | PIK3C2G | NM_004570 | rs7133666 |
| 223 | PIK3C2G | NM_004570 | rs7301521 |
| 224 | PIK3C2G | NM_004570 | rs12309666 |
| 225 | PIK3C2G | NM_004570 | rs17847788 |
| 226 | PIK3C2G | NM_004570 | rs12312266 |
| 227 | PIK3C2G | NM_004570 | rs11044142 |
| 228 | SSPN | NM_005086 | rs34624361 |
| 229 | PRKAG1 | NM_001206709 | rs1126930 |
| 230 | ITGA5 | NM_002205 | rs73322228 |
| 231 | ITGA7 | NM_002206 | rs17117883 |
| 232 | ITGA7 | NM_002206 | rs1800974 |
| 233 | MDM2 | NM_002392 | rs769412 |
| 234 | SGCG | NM_000231 | rs1800350 |
| 235 | SGCG | NM_000231 | rs1800351 |
| 236 | SGCG | NM_000231 | rs17314986 |
| 237 | SGCG | NM_000231 | rs1800353 |
| 238 | ITGBL1 | NM_004791 | rs3916912 |
| 239 | ITGBL1 | NM_004791 | rs2281991 |
| 240 | COL4A1 | NM_001845 | rs3742207 |
| 241 | COL4A1 | NM_001845 | rs874203 |
| 242 | COL4A1 | NM_001845 | rs874204 |
| 243 | COL4A1 | NM_001845 | rs16975492 |
| 244 | COL4A1 | NM_001845 | rs995224 |
| 245 | COL4A2 | NM_001846 | rs4103 |
| 246 | COL4A2 | NM_001846 | rs9583500 |
| 247 | MYH6 | NM_002471 | rs365990 |
| 248 | MYH6 | NM_002471 | rs28711516 |
| 249 | MYH7 | NM_000257 | rs3729830 |
| 250 | MYH7 | NM_000257 | rs7157716 |
| 251 | MYH7 | NM_000257 | rs735712 |
| 252 | MYH7 | NM_000257 | rs2231124 |
| 253 | MYH7 | NM_000257 | rs2069542 |
| 254 | MYH7 | NM_000257 | rs2069540 |
| 255 | ITGA11 | NM_001004439 | rs4777035 |
| 256 | ITGA11 | NM_001004439 | rs2271725 |
| 257 | ITGA11 | NM_001004439 | rs2271724 |
| 258 | ITGA11 | NM_001004439 | rs61729760 |
| 259 | ITGA11 | NM_001004439 | rs2292745 |
| 260 | ITGA11 | NM_001004439 | rs7168069 |
| 261 | ITGA11 | NM_001004439 | rs12907890 |
| 262 | CIITA | NM_000246 | rs4774 |
| 263 | SMG1 | NM_015092 | rs12445870 |
| 264 | SMG1 | NM_015092 | rs56079655 |
| 265 | SMG1 | NM_015092 | rs3816919 |
| 266 | SMG1 | NM_015092 | rs2650613 |
| 267 | ITGAL | NM_002209 | rs1064524 |
| 268 | ITGAL | NM_002209 | rs2230433 |
| 269 | ITGAL | NM_002209 | rs2230434 |
| 270 | ITGAM | NM_000632 | rs11861251 |
| 271 | ITGAM | NM_000632 | rs1143682 |
| 272 | ITGAM | NM_000632 | rs1143683 |
| 273 | ITGAX | NM_000887 | rs2230424 |
| 274 | ITGAX | NM_000887 | rs2230428 |
| 275 | ITGAD | NM_005353 | rs62001041 |
| 276 | MMP2 | NM_001127891 | rs1132896 |
| 277 | MMP2 | NM_001127891 | rs1053605 |
| 278 | MMP2 | NM_001127891 | rs2287074 |
| 279 | MMP2 | NM_001127891 | rs14070 |
| 280 | MMP2 | NM_001127891 | rs11541998 |
| 281 | CIAPIN1 | NM_020313 | rs11557674 |
| 282 | ITGAE | NM_002208 | rs2976230 |
| 283 | ITGAE | NM_002208 | rs3744679 |
| 284 | ITGAE | NM_002208 | rs1183610 |
| 285 | PIK3R5 | NM_001142633 | rs381309 |
| 286 | PIK3R5 | NM_001142633 | rs11650737 |
| 287 | MYH13 | NM_003802 | rs3744550 |
| 288 | MYH13 | NM_003802 | rs17690195 |
| 289 | MYH13 | NM_003802 | rs2074876 |
| 290 | MYH13 | NM_003802 | rs2074877 |
| 291 | MYH13 | NM_003802 | rs2277644 |
| 292 | MYH13 | NM_003802 | rs12938754 |
| 293 | MYH8 | NM_002472 | rs33969260 |
| 294 | MYH8 | NM_002472 | rs3744552 |
| 295 | MYH8 | NM_002472 | rs3744553 |
| 296 | MYH8 | NM_002472 | rs12936716 |
| 297 | MYH8 | NM_002472 | rs34693726 |
| 298 | MYH8 | NM_002472 | rs8068729 |
| 299 | MYH4 | NM_017533 | rs3744554 |
| 300 | MYH4 | NM_017533 | rs11651295 |
| 301 | MYH4 | NM_017533 | rs917361 |
| 302 | MYH4 | NM_017533 | rs3744561 |
| 303 | MYH1 | NM_005963 | rs17811250 |
| 304 | MYH1 | NM_005963 | rs73974725 |
| 305 | MYH1 | NM_005963 | rs2007027 |
| 306 | MYH2 | NM_017534 | rs12600539 |
| 307 | MYH3 | NM_002470 | rs34393601 |
| 308 | MYH3 | NM_002470 | rs2285479 |
| 309 | MYH3 | NM_002470 | rs2285475 |
| 310 | MYH3 | NM_002470 | rs34088014 |
| 311 | MYH3 | NM_002470 | rs2285474 |
| 312 | MYH3 | NM_002470 | rs2285472 |
| 313 | MYH3 | NM_002470 | rs2285469 |
| 314 | MYH3 | NM_002470 | rs876657 |
| 315 | CCL2 | NM_002982 | rs4586 |
| 316 | ITGB3 | NM_000212 | rs5919 |
| 317 | ITGB3 | NM_000212 | rs15908 |
| 318 | ACE | NM_000789 | rs4309 |
| 319 | ACE | NM_001178057 | rs4343 |
| 320 | ITGB4 | NM_001005619 | rs8669 |
| 321 | ACTG1 | NM_001614 | rs1135989 |
| 322 | LAMA1 | NM_005559 | rs2016639 |
| 323 | LAMA1 | NM_005559 | rs671871 |
| 324 | LAMA1 | NM_005559 | rs607230 |
| 325 | LAMA1 | NM_005559 | rs11664063 |
| 326 | LAMA1 | NM_005559 | rs617206 |
| 327 | LAMA1 | NM_005559 | rs12607841 |
| 328 | LAMA1 | NM_005559 | rs62081531 |
| 329 | LAMA1 | NM_005559 | rs16950981 |
| 330 | LAMA1 | NM_005559 | rs62081533 |
| 331 | LAMA1 | NM_005559 | rs12961939 |
| 332 | LAMA1 | NM_005559 | rs12970643 |
| 333 | LAMA1 | NM_005559 | rs73938538 |
| 334 | LAMA1 | NM_005559 | rs662471 |
| 335 | LAMA1 | NM_005559 | rs619106 |
| 336 | LAMA1 | NM_005559 | rs539713 |
| 337 | LAMA1 | NM_005559 | rs684634 |
| 338 | LAMA1 | NM_005559 | rs621993 |
| 339 | LAMA1 | NM_005559 | rs566655 |
| 340 | LAMA3 | NM_001127717 | rs9962023 |
| 341 | LAMA3 | NM_001127717 | rs17202961 |
| 342 | LAMA3 | NM_001127718 | rs1154226 |
| 343 | LAMA3 | NM_001127718 | rs1154232 |
| 344 | LAMA3 | NM_001127718 | rs1131521 |
| 345 | SMAD7 | NM_001190823 | rs3764482 |
| 346 | BMP2 | NM_001200 | rs235768 |
| 347 | BMP2 | NM_001200 | rs13037675 |
| 348 | LAMA5 | NM_005560 | rs875379 |
| 349 | ATP5O | NM_001697 | rs4591 |
| 350 | ATP5O | NM_001697 | rs78234759 |
| 351 | PPARA | NM_001001928 | rs1042311 |
| 352 | DMD | NM_004007 | rs1801187 |
| 353 | COL4A6 | NM_033641 | rs34466065 |
| 354 | COL4A6 | NM_033641 | rs1042065 |
