# Supplementary material for: Tumor Necrosis Factor Receptor SF10A (TNFRSF10A) SNPs Correlate With Corticosteroid Response in Duchenne Muscular Dystrophy
Source: Front Genet. 2020 Jul 3;11:605. doi: 10.3389/fgene.2020.00605 (PMC7350910; doi:10.3389/fgene.2020.00605)
Supplement: TABLE S4 — DAPC analysis SNP output. [file Table_4.doc]

**Table S4. DAPC analysis SNP output.** Discriminant Analysis of Principal Component (DAPC) was performed on: (A) the 134 non-synonymous variants and (B) the 220 synonymous SNPs possibly involved in phenotype modulation in the 21 DiC patients. The analysis sorted out 21 non-synonymous and 22 synonymous SNPs, possibly involved in CS response

**a b**

| CHR | POSITION | GENE | SNP |  | CHR | POSITION | GENE | SNP |
| --- | --- | --- | --- | --- | --- | --- | --- | --- |
| chr8 | 23059324 | TNFRSF10A | rs20575 |  | chr5 | 82789647 | VCAN | rs4470745 |
| chr8 | 23060256 | TNFRSF10A | rs6557634 |  | chr5 | 82786194 | VCAN | rs12332199 |
| chr6 | 1,12E+08 | LAMA4 | rs1050349 |  | chr7 | 20421490 | ITGB8 | rs6968952 |
| chr4 | 1,60E+08 | PPID | rs9410 |  | chr2 | 1,61E+08 | ITGB6 | rs2305818 |
| chr10 | 6002368 | IL15RA | rs2228059 |  | chr7 | 1,08E+08 | LAMB1 | rs11770141 |
| chr2 | 1,14E+08 | IL36A | rs895497 |  | chr7 | 1,08E+08 | LAMB1 | rs25659 |
| chr18 | 6997818 | LAMA1 | rs12961939 |  | chr6 | 1,30E+08 | LAMA2 | rs3749877 |
| chr18 | 46468946 | SMAD7 | rs3764482 |  | chr6 | 1,30E+08 | LAMA2 | rs3749878 |
| chr18 | 6993673 | LAMA1 | rs62081533 |  | chr16 | 18861335 | SMG1 | rs2650613 |
| chr13 | 1,11E+08 | COL4A1 | rs3742207 |  | chr18 | 7011413 | LAMA1 | rs619106 |
| chr12 | 18435452 | PIK3C2G | rs11044004 |  | chr12 | 6138595 | VWF | rs1800380 |
| chr12 | 18649057 | PIK3C2G | rs12312266 |  | chr18 | 6986259 | LAMA1 | rs62081531 |
| chr17 | 79478007 | ACTG1 | rs1135989 |  | chr2 | 2,38E+08 | COL6A3 | rs4433949 |
| chr16 | 57474687 | CIAPIN1 | rs11557674 |  | chr2 | 2,16E+08 | FN1 | rs7596677 |
| chr1 | 46521559 | PIK3R3 | rs785467 |  | chr2 | 2,16E+08 | FN1 | rs1132741 |
| chr18 | 6985270 | LAMA1 | rs11664063 |  | chr2 | 2,16E+08 | FN1 | rs1053238 |
| chr2 | 10908893 | ATP6V1C2 | rs1198849 |  | chr2 | 2,16E+08 | FN1 | rs7589580 |
| chr2 | 2,16E+08 | ATIC | rs2372536 |  | chr16 | 55536727 | MMP2 | rs14070 |
| chr17 | 10355371 | MYH4 | rs11651295 |  | chr2 | 2,28E+08 | COL4A4 | rs2228557 |
| chr17 | 10346781 | MYH4 | rs3744554 |  | chr7 | 1,16E+08 | MET | rs41736 |
| chr17 | 10355763 | MYH4 | rs917361 |  | chr7 | 1,16E+08 | MET | rs2023748 |
|  |  |  |  |  | chr7 | 1,16E+08 | MET | rs41737 |
